# Prussian Blue‐Derived Atomic Fe/Fe_3_C@N‐Doped C Catalysts Supported by Carbon Cloth as Integrated Air Cathode for Flexible Zn‐Air Batteries

**DOI:** 10.1002/advs.202407631

**Published:** 2024-10-28

**Authors:** Zihan Wang, Jing Ren, Guoqiang Ling, Junjie Guo, Yongkang Lv, Rui‐Peng Ren

**Affiliations:** ^1^ State Key Laboratory of Clean and Efficient Coal Utilization Taiyuan University of Technology Taiyuan 030024 China; ^2^ College of Chemistry Taiyuan University of Technology Taiyuan 030024 China; ^3^ Key Laboratory of Interface Science and Engineering in Advanced Materials Ministry of Education Taiyuan University of Technology Taiyuan 030024 China; ^4^ Shanxi‐Zheda Institute of Advanced Materials and Chemical Engineering Taiyuan 030017 China

**Keywords:** Fe_3_C, Fe‐N‐C, oxygen reduction reaction, single atom catalysts, Zn‐air battery

## Abstract

The development of an integrated air cathode with superior oxygen reduction reaction (ORR) performance is fundamental to flexible zinc‐air batteries (ZABs) for wearable electronics. Herein, a self‐assembled metal‐organic framework (MOF)‐derived strategy is proposed to prepare a atomic Fe/Fe_3_C@N‐doped C catalysts supported by carbon cloth (CC) catalyst for use as an air cathode of flexible ZABs. The Prussian Blue precursor, which self‐assembles on the surface of the carbon cloth due to electrostatic attraction, is critical in achieving the uniform dispersion of catalysts with high density loading on carbon cloth substrates. The hollow cubic structure, N‐doped carbon layer coating, and the integrated electrode design can provide more accessible active sites and facilitate a rapid electron transfer and mass transport. Density functional theory (DFT) calculation reveals that the electronic interactions between the Fe‐N_4_ and Fe_3_C dual active sites can optimize the adsorption‐desorption behavior of oxygen intermediates formed during the ORR. Consequently, the Fe/Fe_3_C@N‐doped C/CC exhibits an excellent half wave potential (E_1/2_ = 0.903 V) and superior long‐term cycling stability in alkaline environments. With excellent ORR performance, ZABs and flexible ZABs based on Fe/Fe_3_C@N‐doped C/CC air cathode demonstrate excellent overall electrochemical performance in terms of open circuit voltage, maximum power density, flexibility, and cycling stability.

## Graphical abstract

A MOF self‐assembly and pyrolysis strategy is proposed to prepare Fe/Fe_3_C@N‐doped C/carbon cloth catalysts, in which rapid electron transfer and mass transport are achieved. The electron interaction between Fe single atoms and Fe_3_C active sites lowers the energy barrier, resulting in enhanced ORR catalytic activity. In addition, flexible Zn‐air batteries using the Fe/Fe_3_C@N doped C/carbon cloth air cathode show excellent performance.

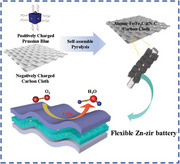

## 1 Introduction

With the booming development of wearable electronics, ZABs, especially flexible ZABs, which are featured by high theoretical energy density, low cost, inherent safety, and environmental friendliness, are regarded as highly competitive energy storage devices for wearable electronics.^[^ ^1^, ^2^, ^3^, ^4^, ^5^, ^6^, ^7^, ^8^, ^9^ ^]^ The actual electrochemical performance of zinc‐air batteries is largely limited by the sluggish kinetics of the ORR during charging/discharging of the air cathode.^[^ ^10^, ^11^ ^]^ Although precious metal catalysts, particularly platinum‐based and ruthenium‐based catalysts, have achieved excellent catalytic performance and have even been used commercially.^[^ ^12^ ^]^ However, the inherent shortcomings, such as high cost due to low reserve and poor stability, have hindered the further development of ZABs.^[^ ^13^, ^14^, ^15^ ^]^

Non‐precious metal based catalysts, especially for the single‐atom catalysts (SACs) with transition metal coordinated with nitrogen anchored on a carbon substrate, are considered the most ideal ORR catalytic alternatives to precious metal catalysts in terms of low cost and competitive electrocatalytic activity.^[^ ^12^, ^16^, ^17^, ^18^, ^19^, ^20^, ^21^, ^22^ ^]^ Among the SACs applied in ORR, the catalysts containing Fe‐N_4_‐C coordination structures demonstrate a preferable catalytic activity and stability due to the more robust structure and the higher intrinsic activity.^[^ ^12^, ^23^, ^24^ ^]^ To date, various synthetic strategies have been developed to prepare single atom Fe‐N_4_‐C catalysts.^[^ ^25^ ^]^ MOF materials are a new class of porous materials with ultra‐high specific surface area, abundant tunable pores, and modifiable surfaces.^[^ ^26^ ^]^ Compared to MOF‐immobilized catalysts, SACs based on MOF derivatives exhibit higher conductivity, ultra‐high thermal and chemical stability, making them more suitable for cathodic ORR.^[^ ^27^ ^]^ Previous reports have clearly demonstrated that MOF‐derived SACs have high catalytic activity and durability for ORR.^[^ ^25^, ^28^, ^29^, ^30^, ^31^, ^32^, ^33^, ^34^, ^35^, ^36^, ^37^, ^38^, ^39^, ^40^ ^]^ To further improve the catalytic activity of single atom Fe‐N_4_‐C catalysts, the Fe‐N_4_‐C electronic structure can be modulated by introducing other neighboring active sites, such as metal carbides, sulfides, nitrides, nanoclusters, and heteroatom‐doping, thereby achieving the optimized energy barriers for ORR.^[^ ^41^, ^42^, ^43^, ^44^ ^]^

However, the majority of SACs containing Fe‐N_4_‐C are in powder form, which requires further preparation of air cathodes by slurry coating, i.e., using conductive agents and binders. The addition of a high percentage of inactive materials will reduce the mass specific capacity and energy density of the air cathode. Moreover, the high tortuosity of these electrodes will increase the resistance to oxygen and electrolyte transport. Despite the addition of binder, the possibility of the catalyst detaching from the substrate during long term charging and discharging is still not negligible, severely limiting the operation lifespan of the powdered catalysts.^[^ ^45^ ^]^ Therefore, the development of integrated flexible electrodes has proven to be an effective strategy to address the above issues. Flexible carbon fiber membrane containing Fe‐N_4_‐C active sites has exhibits excellent ORR performance and surprising durability over 1 000 h.^[^ ^46^ ^]^ Therefore, the assembly of MOF‐derived Fe‐N‐C SACs on flexible substrates, while simultaneously constructing dual active sites to tune the local electronic structure of Fe‐N‐C, is desirable to obtain a high performance flexible integrated air cathode.

Here we have prepared a atomic Fe/Fe_3_C@N‐doped C catalysts supported by carbon cloth for use as an air cathode in flexible ZABs. The Prussian Blue precursor, which self‐assembles on the surface of the carbon cloth due to electrostatic attraction, is critical in achieving the uniform dispersion of catalysts with high density loading on carbon cloth substrates. X‐ray diffractometer (XRD) patterns, high‐angle annular dark‐field scanning transmission electron microscopy (HAADF‐STEM) and X‐ray absorption near‐edge structure (XANES) confirms the the coexistence of the dual active sites of Fe_3_C and atomically dispersed Fe‐N_4_ active sites. We evaluate the ORR performance of the atomic Fe/Fe_3_C@N‐doped C/CC catalysts and further elucidate the effect of the dual active sites on the superior ORR performance by DFT calculations. Finally, we demonstrate the application of the atomic Fe/Fe_3_C@N‐doped C/CC catalysts as an air cathode in aqueous ZABs and flexible ZABs.

## 2 Results and Discussion

**Figure 1:**
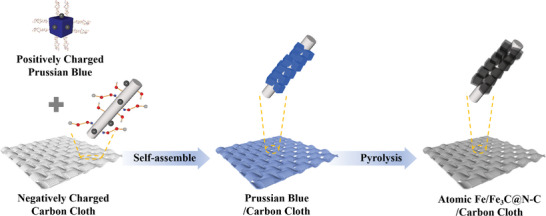
Schematic illustration of the synthesis procedure of the catalyst.

Structure and morphology are key factors for ideal high activity ORR catalysts. The ideal structure with abundant accessible active sites achieves high mass transfer efficiency, resulting in significantly improved electrocatalytic performance. The prepared route of Fe/Fe_3_C@N‐doped C/CC catalysts as shown in **Figure** **1**. Briefly, exposure of CC to high concentrations of nitric acid results in a negatively charged surface due to the formation of carboxyl groups (‐COOH) in an acidic environment. The open three‐dimensional (3D) framework structure accompanied by high porosity and high specific surface area makes Prussian Blue attractive as precursors for electrocatalysts. The colloidal Prussian Blue solution containing the positively charged Prussian Blue crystals, as confirmed by the Tyndall effect (Figure S1, Supporting Information), was synthesized using the cationic surfactant hexadecyl trimethyl ammonium bromide (CTAB). After dipping the negatively charged carbon cloth into the positively charged colloidal Prussian Blue solution, the Prussian Blue will self‐assemble on the surface of the CC due to electrostatic attraction.^[^ ^47^, ^48^, ^49^ ^]^ The Fourier transform infrared (FTIR) spectrum of the acid‐treated CC showed two peaks located at 2341 and 2360 cm^−1^ corresponding to the stretching vibration of C = O, suggesting the presence of the ‐COOH functional group (Figure S2, Supporting Information).

**Figure 2:**
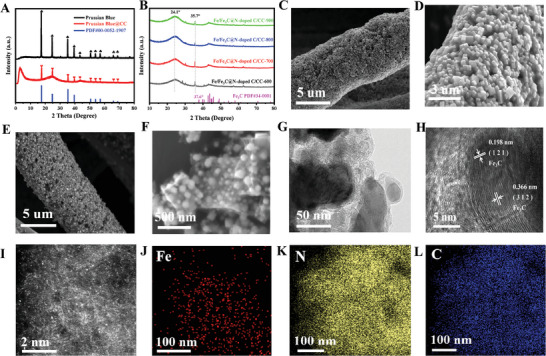
A,B) XRD patterns of as‐prepared samples. SEM images of C,D) Prussian Blue/CC and E,F) Fe/Fe_3_C@N‐doped C/CC. G,H) TEM images of Fe/Fe_3_C@N‐doped C/CC. HAADF image I) and corresponding EDS of J) Fe K) N and L) C elements.

The comparison of the XRD patterns in **Figure** **2**A illustrates that in addition to the characteristic peaks of Prussian Blue (PDF#0052‐1907), the peak at 26° and 44° related to carbon can also be observed in the XRD patterns of Prussian Blue/CC, demonstrating the simultaneous presence of the two components. The peak shift in the XRD pattern is responsible for the mismatch between the diffraction peaks and the standard pattern of Fe_3_C, which reflects the lattice distortion caused by the N‐doping in the carbon material. After pyrolysis, the XRD pattern of Fe/Fe_3_C@N‐doped C/CC contains peaks at ≈24.1° and 43.5°, ascribing to the (002) and (100) facets of CC substrate, respectively. Considering the shift in peak position, the characteristic peak at 35.7° can be attributed to the (121) crystal plane of Fe_3_C (PDF#34‐0001) (Figure 2B). In the pyrolysis stage, the cyanide skeletons of Prussian Blue start to collapse, resulting in phase transformations and the release of carbon nitrides gas fragments. Some of the dissociated Fe bonds with C to form Fe_3_C, and a small amount of Fe forms an Fe‐N coordination with N, which is captured by the nitrogen‐doped carbon layer, resulting in the formation of isolated single iron atoms Fe. After pyrolysis, the final product is atomic Fe/Fe_3_C@N‐doped C/CC. No characteristic peaks associated with Fe were observed in Fe/Fe_3_C@N‐doped C/CC catalyst, which may be due to the high dispersion of Fe in the carbon skeleton at the atomic scale. As observed by scanning electron microscopy (SEM) images (Figures 2C,D and S3, Supporting Information), the cubic Prussian Blue crystals wrap evenly and densely around the entire surface of the carbon fiber of CC, visually demonstrating its high density loading. For comparison, the sample prepared by traditional hydrothermal method shows sparsely distributed Prussian Blue crystals, indicating a low catalyst loading (Figure S4, Supporting Information).

SEM images (Figures 2E,F and S5, Supporting Information) of the final Fe/Fe_3_C@N‐doped C/CC sample show that the Fe_3_C/Fe‐N‐C cubes can still maintain the cube shape of Prussian Blue, which is evenly and densely wrapped around the entire surface of the carbon fiber of CC, greatly increasing the specific surface area of the catalyst and exposing more active sites. A further transmission electron microscopy (TEM) image (Figure 2G) shows more clearly that the Fe/Fe_3_C@N‐doped C cubes have a hollow structure, which are encapsulated by a carbon layer. The hollow structure provides more active sites and a larger electrode/electrolyte contact area, which improves the mass transport efficiency during ORR process. High resolution transmission electron microscopy (HRTEM) image (Figure 2H) shows that the lattice fringes are 0.366  and 0.198 nm, corresponding to the (312) and (121) crystalline plane of Fe_3_C. Figure S6 (Supporting Information) shows that several Fe_3_C particles are anchored on the carbon support. In addition, Fe‐N‐C at the atomic level was further observed by HAADF‐STEM (Figure 2I). The HAADF‐STEM images intuitively show the presence of the abundant Fe single atom sites distributed close to the Fe_3_C, as evidenced by the bright dots. As demonstrated in Figure 2J–L, the elemental mapping result demonstrate that the uniform distribution of Fe, N, and C elements in the Fe/Fe_3_C@N‐doped C structure and the carbon layer is N‐doped, which facilitates the rapid electron transfer during the ORR process. The Fe content in Fe/Fe_3_C@N‐doped C/CC‐700 determined by inductively coupled plasma optical emission spectroscopy (ICP‐OES) was 3.14 wt.% (Tables S1 and S2, Supporting Information). For the samples pyrolyzed at 600 , 800 , and 900 °C, the Fe contents were 3.21, 2.10, and 1.07 wt.%, respectively. The Fe content of the samples obtained at pyrolysis temperatures of 600 and 700 °C was similar. As the pyrolysis temperature was further increased, the Fe content gradually decreased. This can be attributed to the decreased mass loading of Fe/Fe_3_C@N‐doped C on the CC substrate due to the weakened bonding between Fe/Fe_3_C@N‐doped C and the CC substrate at high temperature, which is consistent with the SEM images (Figure S7, Supporting Information).

**Figure 3:**
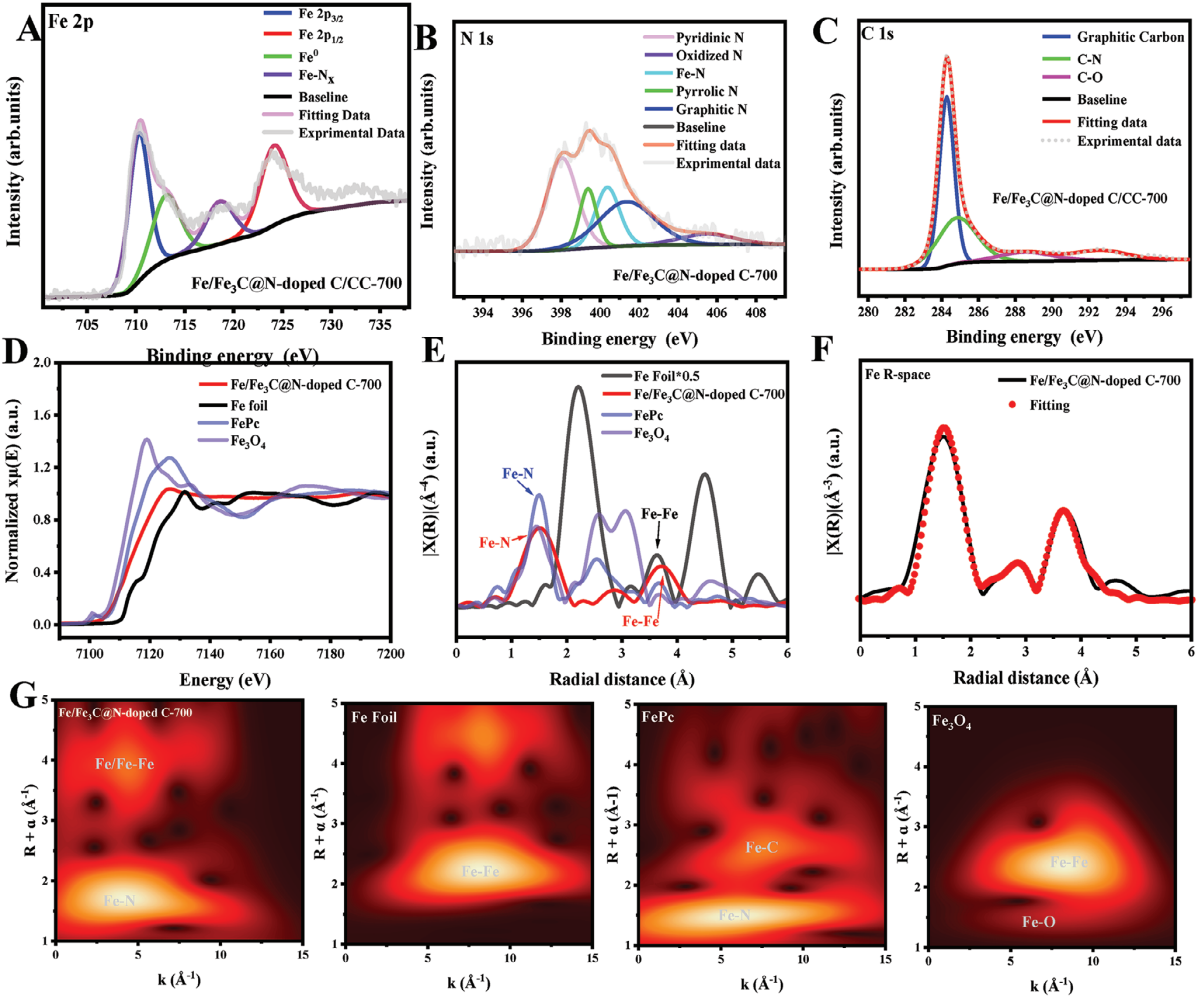
High‐resolution XPS spectra of A) Fe 2p, B) N 1s, and C) C 1s for Fe/Fe_3_C@N‐doped C/CC‐700. D) Fe K‐edge XANES spectra of Fe/Fe_3_C@N‐doped C‐700 and reference samples. E) Fe K‐edge k^3^‐weight FT‐EXAFS spectra. F) Fe K‐edge Fourier transform EXAFS spectrum and its fitting. G) Wavelet transform of the k^3^‐weighted EXAFS data.

The ORR performance of oxygen electrocatalysis is strongly related to the surface properties of the electrocatalysts, so X‐ray photoelectron spectroscopy (XPS) spectra measurements were used to identify the chemical composition and bonding configuration. As shown in **Figure** **3**A, the high‐resolution Fe 2p XPS spectrum of Fe/Fe_3_C@N‐doped C/CC‐700 shows three peaks at 710.28, 712.98, and 718.68 eV belonging to Fe 2p_3/2_, and a peak at 724.28 eV attributed to Fe 2p_1/2_. The strong peak at 712.98 eV belongs to the Fe^0^ species, indicating the presence of Fe_3_C.^[^ ^42^, ^43^, ^44^ ^]^ The peak at 718.68 eV belongs to the single atom Fe‐N_x_ species formed during the pyrolysis process.^[^ ^44^, ^45^, ^46^, ^50^ ^]^ The high‐resolution N 1s spectrum can be divided into five peaks of pyridinic N (397.8 eV), metallic N (399.4 eV), graphitic N (400.9 eV), oxidised N (404.8 eV) and pyrrolic N (399.9 eV)^[^ ^50^, ^51^, ^52^, ^53^ ^]^ (Figure 3B). These configurations are known to be essential for ORR catalysis.^[^ ^54^ ^]^ More importantly, the presence of the metal N peak implies the formation of the Fe‐N coordinate structure in Fe/Fe_3_C@N‐doped C, which is an excellent active site for oxygen electrocatalysis. Furthermore, the C‐N configuration confirmed by the high‐resolution C 1s spectrum suggests successful N‐doping of the carbon (Figure 3C). The Fe K‐edge XANES spectra reveal that the pre‐edge absorption energy of Fe/Fe_3_C@N‐doped C‐700 is between those of Fe foil and Fe_3_O_4_, indicating that the average valance state of Fe is between 0 and +2 (Figure 3D **)**. It is worth noting that the strong characteristic peak of Fe/Fe_3_C@N‐doped C‐700 ≈7130 eV has a negative shift compared to FePc, which makes the actual coordination valence state of Fe in the Fe/Fe_3_C@N‐doped C‐700 structure lower than +2 due to the presence of Fe_3_C.^[^ ^51^ ^]^ The valence and local coordination of Fe in Fe/Fe_3_C@N‐doped C‐700 were further determined by XANES and extended X‐ray absorption fine structure (EXAFS) analyses. As shown in Figure 3E, the Fourier Transform (FT) *k* ^3^‐weighted EXASF spectrum of the Fe K‐edge in the Fe/Fe_3_C@N‐doped C‐700 shows two peaks located at ≈1.47 and 3.74 Å, which are attributed to the Fe‐N and Fe‐Fe scattering pathways, respectively, according to the Fe foil and FePc references. This result verifies the coexistence of atomic level Fe‐N coordination and Fe_3_C. It is worth noting that Fe/Fe_3_C@N‐doped C‐700 has a shortened Fe‐N scattering path compared to FePc as a result of the coupling effect between the Fe_3_C and Fe‐N active sites. As shown in Figures 3F and S8–S10 (Supporting Information), to gain a better understanding of the coordination structure of Fe/Fe_3_C@N‐doped C‐700, EXAFS fitting based on theoretical models was performed on various samples. According to the fitting results, the coordination numbers of Fe‐N and Fe‐Fe in Fe/Fe_3_C@N‐doped C‐700 are close to 4, respectively. As shown in Figure 3G, the wavelet transform (WT) of the *k* ^3^‐weighted EXAFS of the Fe K‐edge was further used to identify the different metal‐N and metal‐metal pathways. The WT contour plots of Fe in the Fe/Fe_3_C@N‐doped C‐700 structure show an intensity maximum around ≈4.5 Å^−1^, which is very similar to that in FePc, further indicating the Fe‐N_4_ coordination in Fe/Fe_3_C@N‐doped C‐700. In addition, Fe/Fe_3_C@N‐doped C‐700 also shows a weak signal at ≈5 Å^−1^, confirming the Fe‐Fe coordination in the Fe/Fe_3_C@N‐doped C‐700 structure. In the XAS spectrum, changes in the coordinating elements could cause a shift in the average bond length due to the coexistence of two coordination structures, Fe‐N and Fe‐Fe. The average bond length in the FT k^3^‐weighted EXAFS spectrum is slightly shifted compared to samples containing only Fe‐N (FePc) or Fe‐Fe (Fe foil). This also demonstrates to some extent the coexistence of Fe‐N_4_ single atom sites and Fe_3_C in the Fe/Fe_3_C@N‐doped C‐700 structure. These findings clearly demonstrate the coexistence of Fe_3_C and atomically dispersed Fe‐N_4_ active sites in the Fe/Fe_3_C@N‐doped C‐700 structure.

**Figure 4:**
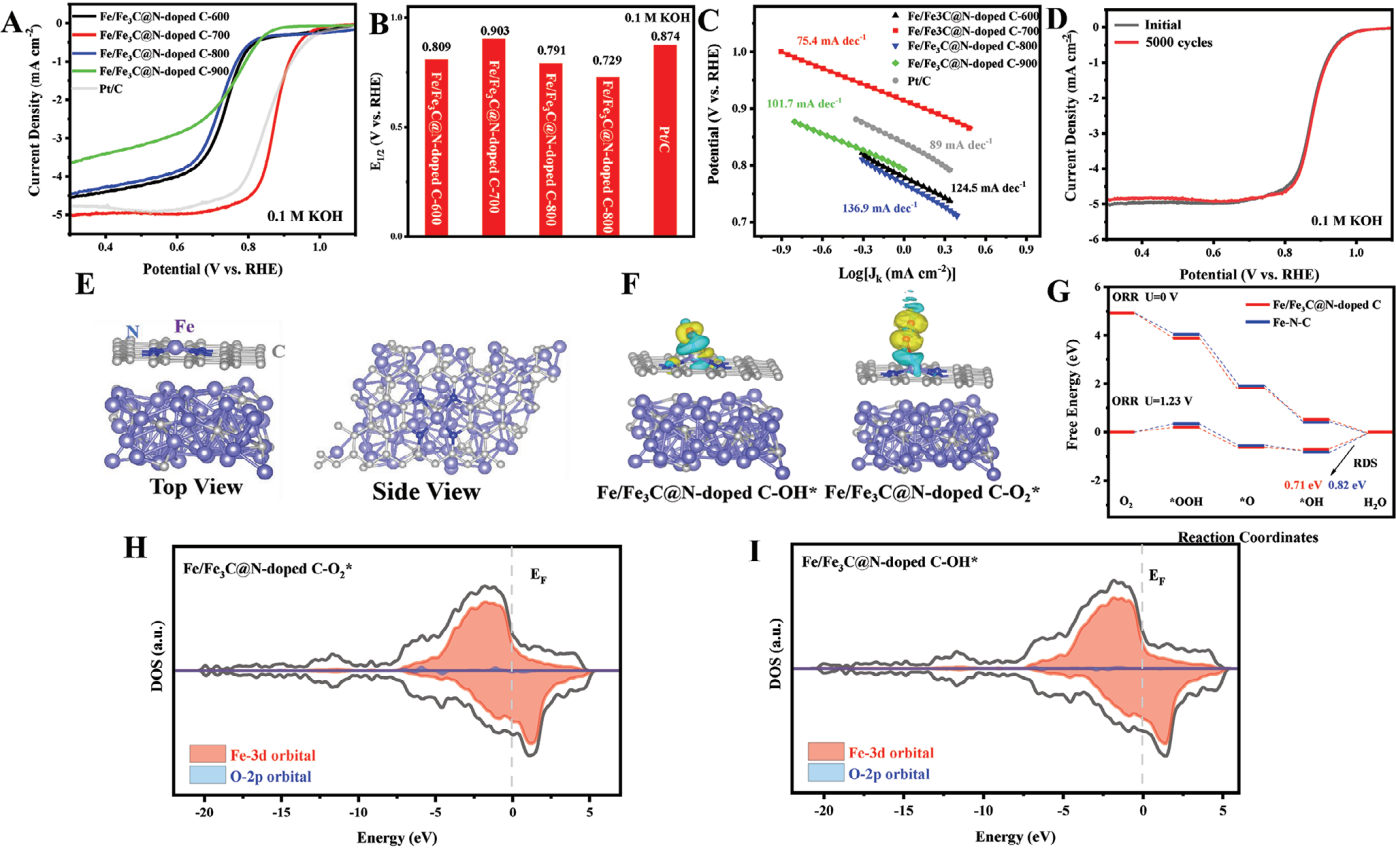
A) LSV curves in the O_2_ saturated 0.1 KOH with the scanning rate of 10 mV s^−1^ at 1600 rpm. B) E_1/2_ performance compare to others samples. C) Corresponding Tafel plots. D) Stability test before and after 5000 potential cycles. E) Computational Structure Model of Fe/Fe_3_C@N‐doped C. F) Electron difference density plots of O_2_* and OH* intermediate adsorption. G) ORR Gibbs free energy diagrams of Fe/Fe_3_C@N‐doped C and Fe‐N‐C at U = 0 V and 1.23 V. H) and I) Fe/Fe_3_C@N‐doped C for DOSs of O_2_* and OH* intermediates, where the Fermi level is represented by a gray dashed line.

The air cathode with excellent ORR activity and stability are essential for high performance ZABs. The ORR test of the as‐prepared catalyst was performed using rotating disc electrode (RDE) voltammetry in an oxygen‐saturated 0.1 M KOH electrolyte. A commercially available Pt/C oxygen reduction catalyst was used as a reference catalytic electrode. The cyclic voltammetry (CV) curves show that a peak appeared in the O_2_ saturated electrolyte and was absent in the N_2_ saturated electrolyte, indicating the good catalytic activity of the Fe/Fe_3_C@N‐doped C/CC catalyst without side reactions (Figure S11, Supporting Information). High temperature pyrolysis can cause the 3D framework structure to collapse, leading to metal agglomeration, which in turn results in a significant reduction in the number of active sites, reducing catalytic performance. It is therefore necessary to optimize the pyrolysis temperature in order to obtain highly active catalysts. As shown in **Figure** **4**A, Linear sweep voltammetry (LSV) curves demonstrate that the Fe/Fe_3_C@N‐doped C/CC‐700 has a high oxygen reduction activity, with a half‐wave potential (E_1/2_) value of 0.903 V versus RHE and a one‐set potential (E_onset_) value of 1.04 V versus RHE, which is competitive and superior to previously reported ORR catalysts (Figure 4B; Table S3 and S4, Supporting Information). In addition, the Fe/Fe_3_C@N‐doped C/CC‐700 has the smallest Tafel slope (75.4 mA dec^−1^) than Fe/Fe_3_C@N‐doped C/CC‐600 (101.7 mA dec^−1^), Fe/Fe_3_C@N‐doped C/CC‐800 (136.9 mA dec^−1^), Fe/Fe_3_C@N‐doped C/CC‐900 (124.5 mA dec^−1^), and commercial Pt/C catalysts (92 mA dec^−1^), implying the excellent ORR kinetic activity (Figure 4C). ORR durability is an important indicator of oxygen electrocatalysts. The voltage of the accelerated stability test was between 0.2 and 1.2 V versus RHE. The Fe/Fe_3_C@N‐doped C/CC‐700 shows impressive durability in alkaline environments, with virtually no drift after 5000 cycles and no significant change in E_1/2_ (Figure 4D). The high loading of Fe‐based active sites combined with the synergistic effects between the Fe‐N_4_‐C and Fe_3_C species were responsible for the superior ORR catalytic activity and durability of Fe/Fe_3_C@N‐doped C/CC‐700.^[^ ^51^, ^53^, ^55^ ^]^ The oxygen evolution reaction performance of the Fe/Fe_3_C@N‐doped C/CC‐700 catalyst was also studied in O_2_‐saturated 0.1 M KOH. With a mass loading of 0.71 mg cm^−2^, the Fe/Fe_3_C@N‐doped C/CC‐700 gives an overpotential of 416.3 mV at 10 mA cm^−2^ (Figure S12, Supporting Information).

First‐principles‐based DFT was used to construct Fe_3_C exposed (1 2 1) crystal planes and adjacent Fe‐N‐C structures, further revealing the mechanism of the interface effect caused by Fe_3_C in Fe‐N‐C structures on the overall ORR activity (Figure 4E). As a comparison, the Fe‐N‐C model was also constructed (Figure S13, Supporting Information). The relevant mechanism steps of ORR reaction are shown in Equations (1)–(4) (* only represents the active sites on the catalyst):

(1) $$O_{2}\left(g\right)+*+H_{2}O+e^{-}=*\mathrm{OOH}+OH^{-}$$

(2) $$*\mathrm{OOH}+e^{-}=*O+OH^{-}$$

(3) $$*O+H_{2}O+e^{-}=\mathrm{OH}*+OH^{-}$$

(4) $$*\mathrm{OH}+e^{-}=*+OH^{-}$$

Based on the aforementioned experiments, we speculate that the interface effect caused by introducing Fe_3_C into the original spatial structure of Fe‐N‐C can to some extent reduce the potential energy barrier on the active sites of Fe‐N‐C during the ORR process. In this process, the adsorption and desorption of O* and OH* active intermediates have always been considered as the rate determining step of the entire ORR reaction. To verify this hypothesis, as illustrated in Figures 4F and S14 (Supporting Information), we constructed a differential density charge model for the adsorption and desorption of O* and OH* active intermediates by Fe/Fe_3_C@N‐doped C. Compared to the Fe‐N‐C structure (Figures S15 and S16, Supporting Information), Fe_3_C, as an electron rich body, transfers electrons to the electron deficient Fe‐N‐C structure (Figure S17, Supporting Information). Thus, electron donation from Fe_3_C is likely to modulate the adsorption‐desorption of O‐related intermediates on the Fe‐N_4_‐C sites, resulting in the enhanced ORR performance. The rate of a reaction determines the energy barrier size of the step, which often represents the difficulty of the reaction occurring. In order to reveal the RDS and related energy barrier sizes, Gibbs free energy diagrams were constructed for the ORR process of Fe/Fe_3_C@N‐doped C and Fe‐N‐C models under the four electron mechanism (Figure 4G). Under the condition of 0 V, both models exhibit consistent downhill energy processes, indicating the existence of spontaneous thermodynamic exothermic processes. At a potential of 1.23 V, the highest Gibbs free energy difference between the two models appears in the process of OH* + e^−^ = * + OH^−^, indicating that this step is a RDS. The RDS energy barriers of each model are Fe/Fe_3_C@N‐doped C (0.71 eV) and Fe‐N‐C (0.82 eV), respectively. This result confirms the hypothesis and jointly indicates that Fe_3_C can effectively enhance the ORR kinetics on the Fe‐N‐C active site. As shown in Figure 4H,I, density of states (DOSs) of O* and OH* active intermediates on Fe/Fe_3_C@N‐doped C was constructed to further reveal the electronic effect of bond strength. Compared to the Fe‐N‐C model (Figures S18 and S19, Supporting Information), the overlap between the Fe 3d and O 2p orbitals of O* active intermediates is smaller, while the overlap between the orbitals of OH* active intermediates is greater. This indicates that Fe/Fe_3_C@N‐doped C has a better adsorption effect on O_2_ and a lower desorption energy barrier for OH* active intermediates. Thus, the introduction of Fe_3_C into the Fe‐N‐C structure can reduce the ORR active energy barrier, optimize the adsorption‐desorption process of O* and OH* intermediates, and enable Fe/Fe_3_C@N‐doped C to have excellent ORR activity.

**Figure 5:**
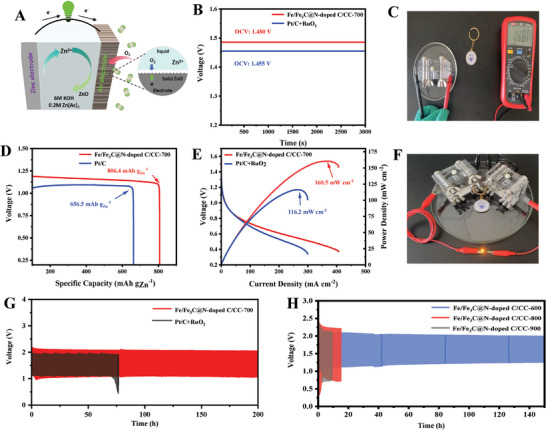
A) Customized mold schematic diagram. B) The initial OCV. C) Voltage measured by a multimeter. D) Specific discharging capacities of the batteries under 10 mA cm^−2^. E) Discharge polarization curves and power density curves. F) ZABs successfully lights up the LED screen. Galvanostatic cycling stabilities under 10 mA cm^−2^ charge and discharge of G) Fe/Fe_3_C@N‐doped C/CC‐700 and H) Fe/Fe_3_C@N‐doped C/CC‐600, Fe/Fe_3_C@N‐doped C/CC‐800, Fe/Fe_3_C@N‐doped C/CC‐900, respectively.

Motivated by the outstanding ORR performance of Fe/Fe_3_C@N‐doped C/CC, aqueous rechargeable Zn‐air batteries (ZABs) were assembled to demonstrate their practical performance (**Figure** **5**A). For comparison, a commercial 20 wt.% Pt/C and RuO_2_ mixture (mass ratio 1:1) was prepared as the air cathode. The Fe/Fe_3_C@N‐doped C/CC‐700 based ZAB showed a high open circuit voltage (OCV) of 1.480 V, which is slightly higher than 1.455 V for the reference Pt/C+RuO_2_ based batteries (Figure 5B,C). The Fe/Fe_3_C@N‐doped C/CC‐700 air cathode assembled ZAB demonstrates a high specific capacity of 806.4 mA g_Zn_^−1^ at 10 mA cm^−2^ and the resulting maximum power density of 160.5 mW cm^−2^, superior to the reference Pt/C+RuO_2_ based ZAB (1.455 V, 656.5 mA g_Zn_^−1^ and 116.2 mW cm^−2^, respectively) (Figure 5D,E). To directly demonstrate the potential application of Fe/Fe_3_C@N‐doped C/CC‐700 air cathode assembled ZAB, we connected two ZABs in series to power a white LED lamp (Figure 5F). The Fe/Fe_3_C@N‐doped C/CC‐700 based battery could operate well over 200 h (600 cycles) at 10 mA cm^−2^ for 10 min charge/discharge (Figure 5G), which is considerably longer than other Fe/Fe_3_C@N‐doped C/CC catalysts obtained at different pyrolysis temperatures (Figures 5H and S20, Supporting Information) and reference Pt/C + RuO_2_ assembled ZAB (less than 80 h). It is worth noting that Fe/Fe_3_C@N‐doped C/CC‐600 has similar ORR properties to Fe/Fe_3_C@N‐doped C/CC‐800, but their ZABs performance is significantly different. For the ZAB performance measurement, our flexible Fe/Fe_3_C@N‐doped C/CC catalyst was used directly as an air cathode. Thus, the ZAB performance depends on both the ORR performance and the actual mass loading of Fe/Fe_3_C@N‐doped C on the CC substrate. With excellent ORR performance and high mass loading of ∼0.8 mg cm^−2^, ZABs based on Fe/Fe_3_C@N‐doped C/CC‐700 air cathode have excellent overall electrochemical performance in terms of OCV, specific capacity, maximum power density, and cycling stability.

**Figure 6:**
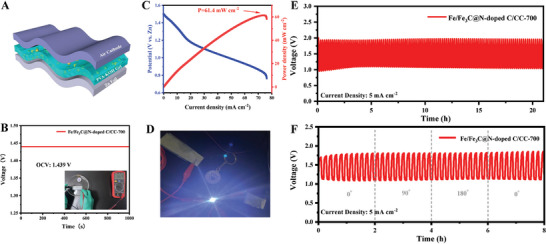
Quasi‐solid‐state flexible ZABs performance based on Fe/Fe_3_C@N‐doped C/CC‐700. A) FZABs schematic diagram. B) OCV test and measured by a multimeter. C) The corresponding power density curves. D) Successfully lights up the LED. (E‐F) Long‐term cycling performance.

Flexible zinc air batteries (FZABs) can be used to power flexible and portable electronic devices due to their high energy density, intrinsic safety, and flexible mechanical properties. Thanks to the flexibility of the CC substrate, the Fe/Fe_3_C@N‐doped C/CC‐700 can be used directly for the assembly of FZAB (**Figure** **6**A), which exhibits a stable OCV of 1.439 V within 1000 s and voltage measured by a multimeter (Figures 6B and S21, Supporting Information). The corresponding power density shows a high peak power density of 61.4 mW cm^−2^ at the current density of 75.8 mA cm^−2^ (Figure 6C). The long term stability is a key factor for the practical application in a wearable electronic device. Considering the practical application of quasi‐solid‐state FZAB, a serial connection of two Fe/Fe_3_C@N‐doped C/CC‐700 based quasi‐solid‐state FZABs successfully powers an LED (Figure 6D). As depicted in Figure 6E, it showed a stable operation of over 21.5 h at a current density of 5 mA cm^−2^ for 10 min charge/discharge, which is better than flexible Fe/Fe_3_C@N‐doped C/CC air cathodes obtained at different pyrolysis temperature (Figures S22 and S23, Supporting Information). In addition, we examined the mechanical flexibility of the designed FZAB for use in wearable electronics. The FZAB exhibited a stable charge/discharge voltage at different bending states (Figure 6F), and the bounding condition can be seen in Figure S24 (Supporting Information). The FZABs assembled by Fe/Fe_3_C@N‐doped C/CC‐700 air cathode exhibited high OCV, maximum power densities, long lifespan, and excellent mechanical flexibility.

## 3 Conclusion

In summary, we have developed a self‐assembled MOF‐derived strategy to prepare an integrated Fe/Fe_3_C@N‐doped C/CC catalyst with dual active sites of Fe‐N_4_ and Fe_3_C for use as an air cathode in ZABs. The Prussian Blue precursor, which self‐assembles on the surface of the carbon cloth due to electrostatic attraction, is critical in achieving the high density loading of catalysts on carbon cloth substrates. The hollow structure of final catalyst provides more active sites and a larger electrode/electrolyte contact area, which improves the mass transport efficiency during ORR process. Moreover, the outer N‐doped C layer and the integrated electrode design both contribute to the rapid electron transfer during the ORR process. DFT calculations demonstrate that the dual active sites exhibit a higher electron transfer density between the oxygen intermediate and the catalyst, reduce the energy barrier of the reaction‐determining steps, and favor the adsorption‐desorption processes of active intermediates such as O* and OH*. As a result, the Fe/Fe_3_C@N‐doped C/CC exhibits an excellent half wave potential (E_1/2_ = 0.903 V) and superior long‐term cycling stability in alkaline environments. The Fe/Fe_3_C@N‐doped C/CC‐700 is directly used as the air cathode of the rechargeable ZAB with an OCVs of 1.480 V, a high specific capacity of 806.38 mAh g_Zn_^−1^ and excellent cycling stability over 200 h at 10 mA cm^−2^, and a peak power density of 160.5 mW cm^−2^, significantly superior to the commercial Pt/C+RuO_2_ catalysts. Furthermore, when equipped as a rechargeable FZAB, it demonstrated an OCVs of 1.439 V, a peak power density of up to 61.4 mW cm^−2^, and excellent mechanical flexibility. This work demonstrates that rational structure and component design can effectively improve the activity of catalysts, which may provide insights into high‐performance flexible air cathodes for ZABs.

## Conflict of Interest

The authors declare no conflict of interest.

## Supporting information



Supporting Information

## Data Availability

The data that support the findings of this study are available from the corresponding author upon reasonable request.
