# Supplementary material for: Prussian Blue‐Derived Atomic Fe/Fe3C@N‐Doped C Catalysts Supported by Carbon Cloth as Integrated Air Cathode for Flexible Zn‐Air Batteries
Source: Adv Sci (Weinh). 2024 Oct 28;12(1):2407631. doi: 10.1002/advs.202407631 (PMC11714216; doi:10.1002/advs.202407631)
Supplement: Supplementary file 1 — Supporting Information [file ADVS-12-2407631-s001.docx]

**Supporting Information**

**Prussian Blue-Derived Atomic Fe/Fe_3_C@N-doped Catalysts Supported by Carbon Cloth as Integrated Air Cathode for Flexible Zn-Air Batteries**

Zihan Wang^a^, Jing Ren^b*^, Guoqiang Ling^b^, Junjie Guo^c*^, Yongkang Lv^a,d^, Rui-Peng Ren^a,d*^

1. State Key Laboratory of Clean and Efficient Coal Utilization, Taiyuan University of Technology, Taiyuan 030024, China.
2. College of Chemistry, Taiyuan University of Technology, Taiyuan 030024, China.
3. Key Laboratory of Interface Science and Engineering in Advanced Materials, Ministry of Education, Taiyuan University of Technology, Taiyuan 030024, China
4. Shanxi-Zheda Institute of Advanced Materials and Chemical Engineering, Taiyuan 030017, China.

**Email**: renjing02@tyut.edu.cn (Jing Ren); [guojunjie@tyut.edu.cn](mailto:guojunjie@tyut.edu.cn) (Junjie Guo); [renruipeng@tyut.edu.cn](mailto:renruipeng@tyut.edu.cn) (Rui-Peng Ren)

**Experimental Section**

**Materials**

Cobalt(Ⅱ) nitrate hexahydrate, polyvinylpyrrolidone (PVP, K-30), poly(vinyl alcohol) 1788 (PVA), hexadecyltrimethylammonium bromide (CTAB), concentrated nitric acid (HNO_3_), concentrated hydrochloric acid (HCl) were purchased from sinopharm chemical reagent Co., Ltd (China). 2-Methylimidazole (C_4_H_6_N_2_), citric acid monohydrate (C_6_H_10_O_8_) were purchased from Aladdin (China). Potassium ferrocyanide trihydrate (K_4_FeC_6_N_6_H_6_O_3_) was purchased from Sigma-Aldrich (America). Carbon cloth (CC) was purchased from Shanghai Jingchong Electronic Technology Development Co., Ltd (China). Carbon paper (CP) was purchased from Toray Industries, Inc (Japan). Anhydrous ethanol (C_2_H_5_OH, ≥99.7%) was purchased from Tianjin Damao Chemical Reagent Factory (China). Nafion (5 wt%) from Shanghai Chuxi Industrial Co., Ltd (China). Pt/C (20 wt%) and RuO_2_ were purchased from Alfa Aesar (America). All chemicals were used as received without further purification.

**Synthesis of Prussian Blue powder**

Briefly, 7.6 g PVP K-30, 0.22 g K_4_[Fe(CN)_6_]·3H_2_O and 0.5134 g C_6_H_8_O_7_·H_2_O were dissolved in 90 ml 0.1 M HCl solution and stirred for at least 30 h until the colour of the solution changed from light yellow to dark green. The solution was then transferred to a 100 ml Teflon-lined autoclave and kept at 80 ℃ for 24 h to form Prussian blue. The precipitate was collected by centrifugation and then washed several times with ethanol until the supernatant became clear. Finally, the prepared product was vacuum treated at 60 ℃ for 12 h and completely ground for further use.

**Synthesis of CTAB functionalized Prussian Blue solution**

Firstly, 100 mg Prussian Blue powder was dispersed into 10 ml 0.06 mg·ml^-1^ CTAB solution with the help of ultrasonic to form the homogenous Prussian Blue solution.

**Self-assembly of MOFs on carbon cloth**

The carbon cloth was soaked in high concentrated HNO_3_ for at least 24 h under dark condition to obtain the carbon cloth or carbon paper with negatively charged surface, and washed with ultra-pure water to remove the extra high concentrated HNO_3_. Finally, the acid-treated carbon cloth or carbon paper was dried under 60 ℃ overnight. The acid-treated carbon paper or cloth was soaked in the precursor solution for at least 1 h using ultrasound, then pulled out and absorbent cotton was used to absorb the excess solution. After drying for at least 1 h under the condition of 60 ℃ in an electric oven, the carbon cloth or carbon paper was soaked in the Prussian blue solution again and the same procedure was repeated two more times. The self-assembled MOFs on the carbon cloth or carbon paper were finally finished and named as Prussian Blue/CC. For comparison, a conventional hydrothermal method was used to synthesis Prussian Blue/CC. Briefly, the commercial carbon cloth or carbon paper was added directly into the Teflon-lined autoclave with the dark green solution, kept at 80 ℃ for 24 h and washed with ethanol several times. Finally, the hydrothermal product was vacuum treated at 60 ℃ for 12 h for further use and named Prussian Blue/CC-Hydro.

**Synthesis of Fe/Fe_3_C@N-doped C/CC-700 electrode**

The Prussian blue/CC was heated at 700 ℃ with a heating rate of 5 ℃·min^-1^ for 2 h under the N_2_ flow rate of 30 ml·min^-1^. The resulting electrode was named Fe/Fe_3_C@N-doped C/CC-700. To investigate the effect of different temperatures on the catalytic performance, the different temperatures were named as Fe/Fe_3_C@N-doped C/CC-600, Fe/Fe_3_C@N-doped C/CC-800 and Fe/Fe_3_C@N-doped C/CC-900, respectively.

**Fabrication of Zinc-air battery**

For the liquid electrolyte based zinc-air battery test, the Fe/Fe_3_C@N doped C/CC-700 electrodes were used as the cathode and zinc foil as the anode, while 6 M KOH and 0.2 M Zn(CH_3_COO)_2_ solution were selected as the electrolyte. All materials were assembled in the custom mould. To assemble the flexible zinc-air battery, 3 g of poly(vinyl alcohol) (PVA) was added to 20 ml of ultra-pure water heated to 80 ℃ in an oil bath with vigorous stirring until the PVA powder was completely dissolved. Then, the 10 ml of 0.3 g·min^-1^ KOH was added to the PVA solution to prepare the gel alkaline poly(vinyl alcohol) (PVA) membrane as electrolyte. Other materials were the same as for the liquid electrolyte zinc-air battery.

**Materials characterization**

A scanning electron microscope (SEM) was used to investigate the morphology on a ThermoFisher Quattro SEM. X-ray diffraction (XRD) on the Rigaku Ultima IV diffractometer with Cu Kɑ X-rays toidentify the crystalline phases. The Fe content was measured by inductively coupled plasma atomic emission spectroscopy (ICP-OES) (Agilent 5110). The sample quality change under different temperature was measured by thermogravimetric Analysis (TG) (NETZSCH, STA 449 F3). X-ray photoelectron spectroscopy (XPS) was performed using a Thermo Scitenfic K-Alpha equipped with a hemispherical energy analyzer and a monochromatic Al Kɑ source. A Renishaw Raman system was used to perform Raman spectroscopy and analyze the degree of graphitization of materials under excitation at 514 nm and 325 nm. Transmission electron microscopy (TEM, FEI Talos F200X) and high resolution TEM (HRTEM, FEI Talos F200X) were used to further observe the surface structure of catalysts. High-angle circular dark field scanning TEM (HAADF-STEM, Jeol JEM-ARM300F GRAND ARM) was used to prove the existence of single atom. The X-ray absorption near edge structure (XANES) and the extended X-ray absorption fine structure (EXAFS) tests of Ni and Fe K-edge of the catalysts were carried out at the XAFCA beamline of Shanghai Synchrotron Radiation Facility (SSRF) in a transmission mode.

**Electrochemical measurements**

The electrochemical test were performed on a CHI660e electrochemical workstation (Shanghai Chenhua Co., China) in typical three-electrode system at standard temperature and pressure condition. The glassy carbon electrode (5mm in diameter) as working electrode. A graphite rod (6mm in diameter) was selected as counter electrode, and saturated calomel electrode (SCE, filled with saturation KCl solution) was selected as reference electrode, all potential values in this work were reported versus reversible hydrogen electrode (RHE) by equation:

E_RHE_ = E_SCE_ + 0.059 × pH + 0.241.

After removing the Fe/Fe_3_C@N-doped C catalyst from CC through ultrasound,5 mg of prepared catalyst was dispersed into 200 μL ethanol, 190 μL water, and 10 μL 5wt.% Nafion was subjected to ultrasound for 60 minutes to prepare homogeneous catalyst ink. Drop 10ul of catalyst ink prepared onto a clean glassy carbon electrode and air dry naturally to form a thin film, resulting in the catalysts loading of 0.3822 mg cm^-2^, was prepared in the same way in 20 wt.% Pt/C. All the tests were conducted in 0.1 M KOH solutions and O_2_ or N_2_ was ventilated for 0.5 h to ensure the saturation of electrolyte before the test. The cyclic voltammogram (CV) curves under N2 and O2 saturation were tested at a scan rate of 10 mV s^-1^. The linear sweep voltammogram (LSV) tests were recorded at the speed range of 1600 rpm at a scan rate of 10 mV s^-1^.

The home-made Zn-air batteries (ZABs) were assembled using polished zinc foil as the anode and aqueous solution containing 6 mol L^-1^ KOH + 0.2 mol L^-1^ zinc acetate as the electrolyte and flexible Zn-air batteries (FZABs). Fe_3_C/Fe-N-C/CC directly as the air cathode, and commercial Pt/C+RuO_2_ (with a mass ration of 1:1) coated on carbon paper (repeatedly adding drops multiple times to ensure the catalyst loading is consistent with Fe/Fe_3_C@N-doped C/CC The load capacity is the same) was used as the air cathode in customized molds. In addition, in the FZABs system, KOH-PVA was selected as electrolyte, another condition were same with ZABs system. The galvanostatic charge and discharge curves were recorded using a Neware battery testing station (CT-4008Tn) at room temperature for a cycling interval of 20/10 mins (10/5 mins for discharging and 10/5 mins for charging).

**DFT Calculation**

All the DFT calculations were conducted based on the Vienna Ab-inito Simulation Package (VASP)^[1-2]^. The exchange-correlation effects were described by the Perdew-Burke-Ernzerhof (PBE) functional within the generalized gradient approximation (GGA) method^[3-4]^. The core-valence interactions were accounted by the projected augmented wave (PAW) method^[5]^. The energy cutoff for plane wave expansions was set to 480 eV, and the 3×3×1 Monkhorst-Pack grid k-points were selected to sample the Brillouin zone integration. The vacuum space is adopted 15 Å above the surfaces to avoid periodic interactions. The structural optimization was completed for energy and force convergence set at 1.0×10^-4^ eV and 0.02 eV Å^-1^, respectively.


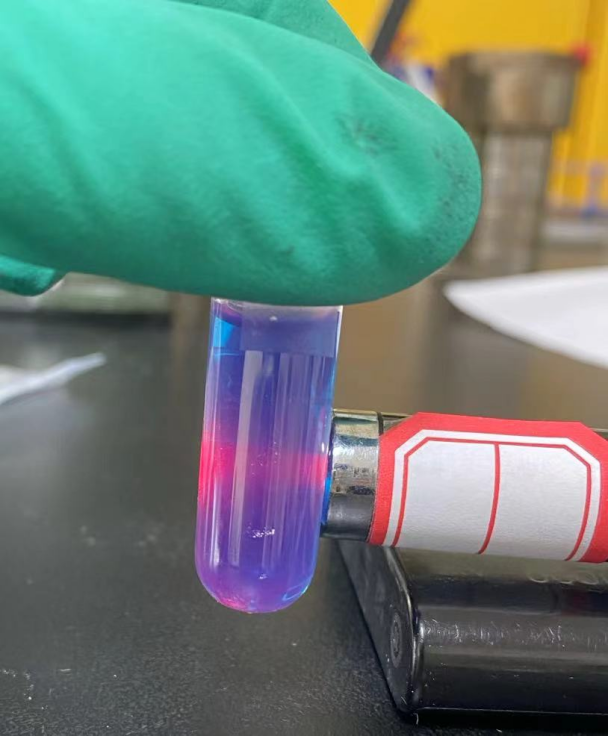


**Figure S1.** Tyndall effect of Prussian Blue Colloidal Solution.


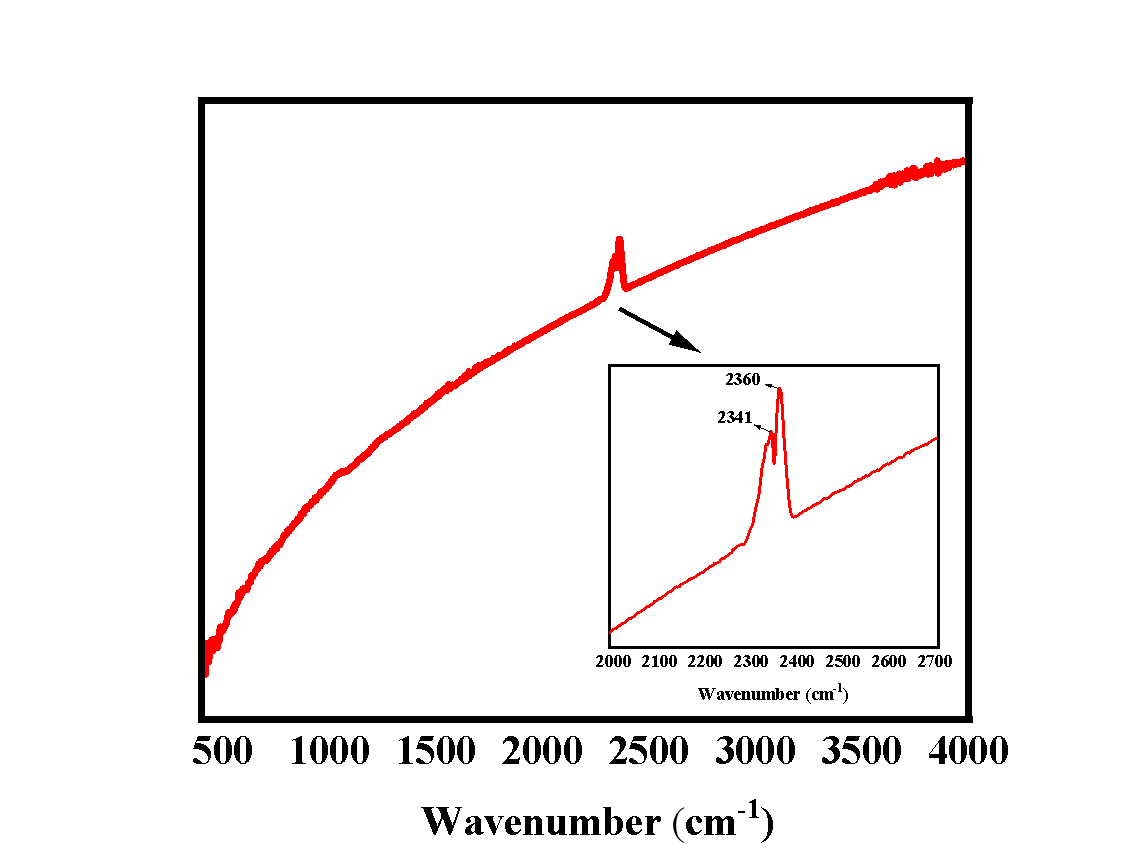


**Figure S2.** FTIR spectra of the acid-treat carbon cloth.

**
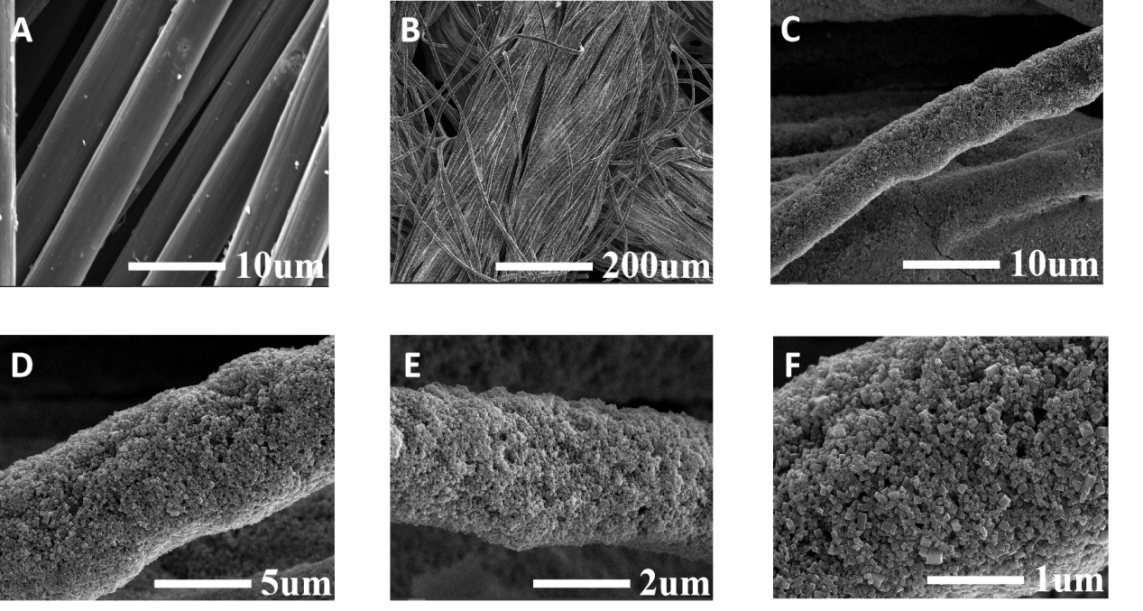
**

**Figure S3.** SEM image of A) CC and B-F) Prussian Blue/CC.


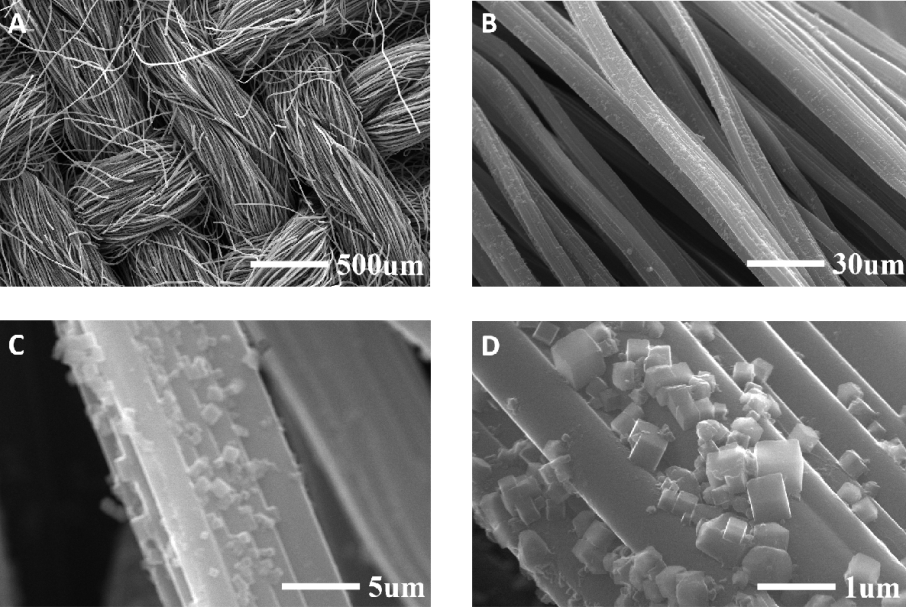


**Figure S4.** SEM images of Prussian Blue/CC-Hydro.


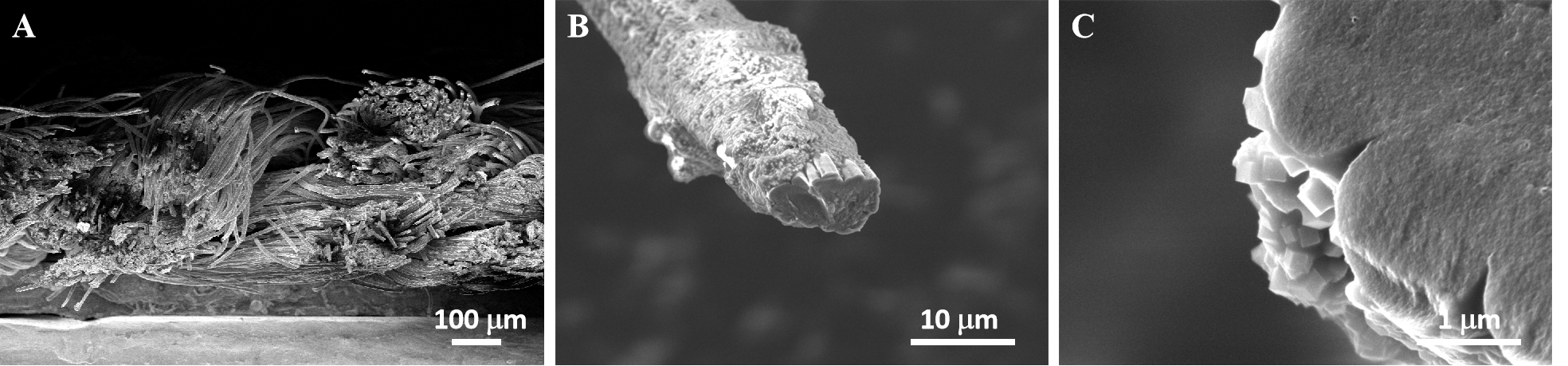


**Figure S5.** Cross-sectional SEM image of Fe/Fe_3_C@N-doped C/CC.


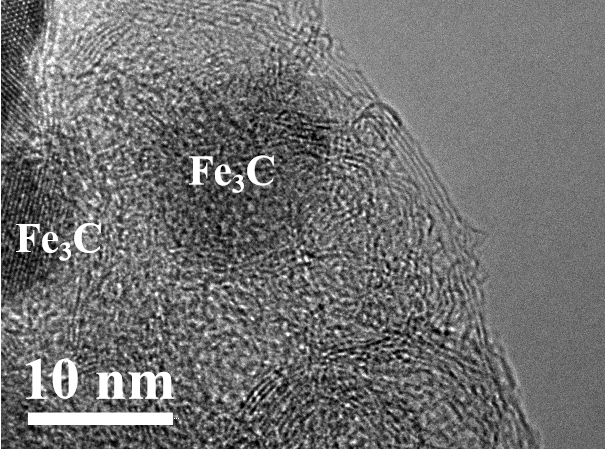


**Figure S6.** TEM image of Fe/Fe_3_C@N-doped C/CC.


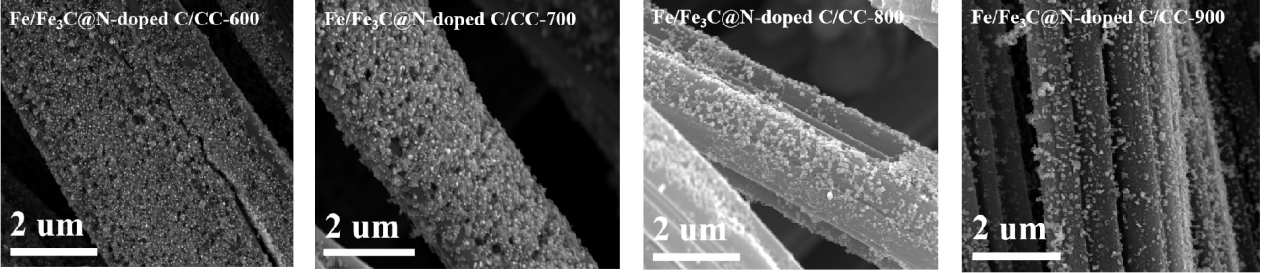


**Figure S7.** SEM image of Fe/Fe_3_C@N-doped C/CC at pyrolysis temperatures.


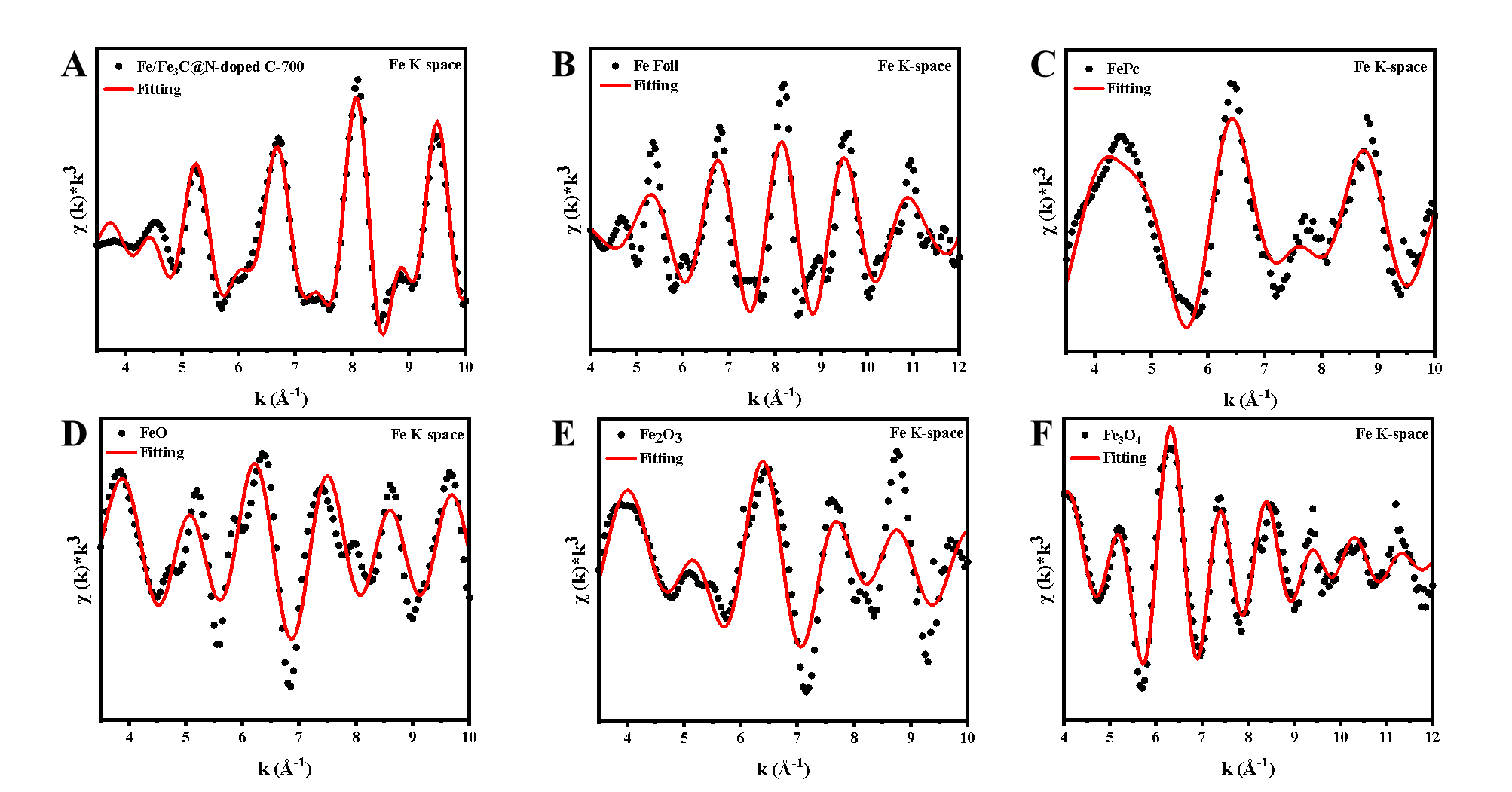


**Figure S8.** EXAFS Fitting in K-space.


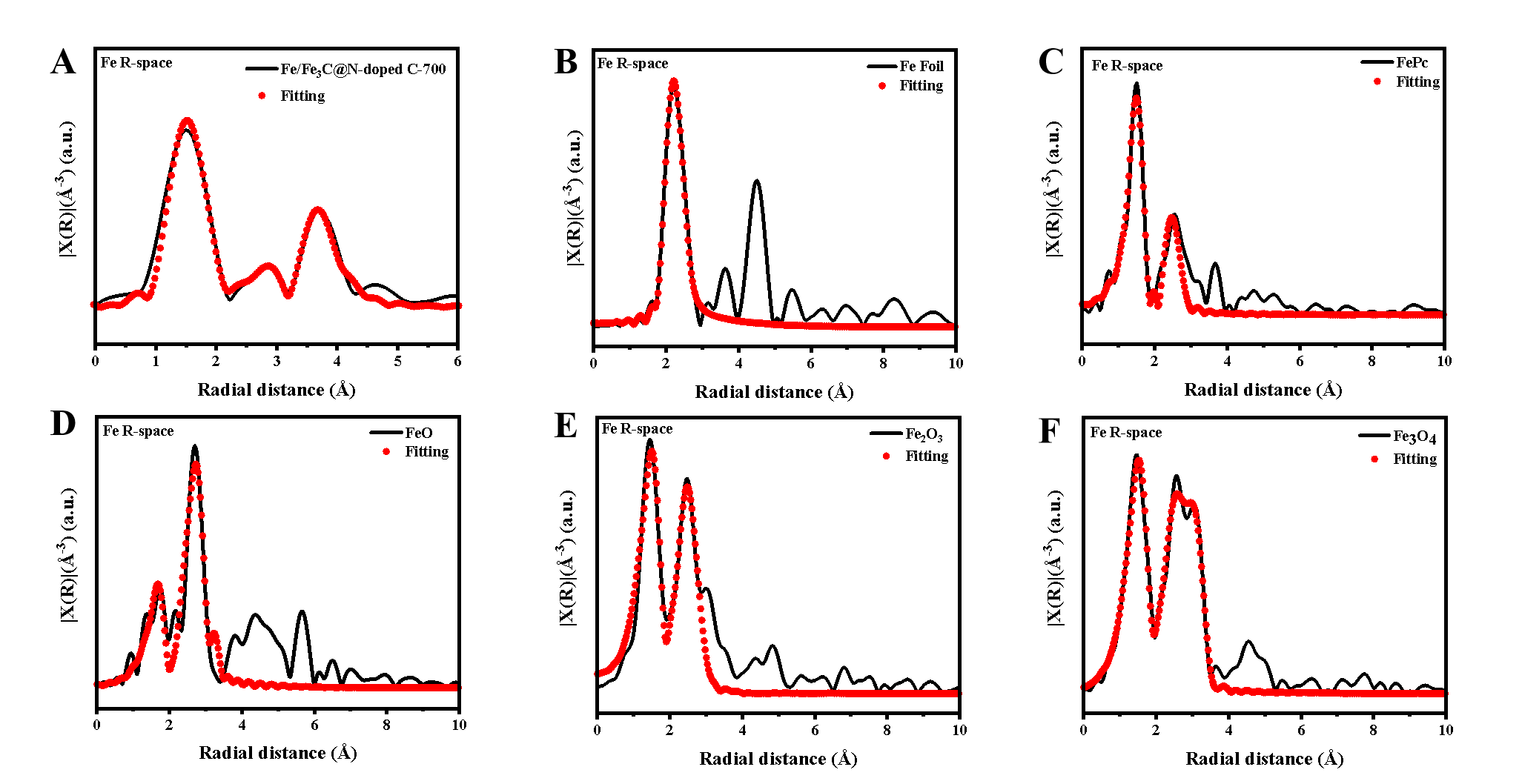


**Figure S9.** EXAFS Fitting in R-space.


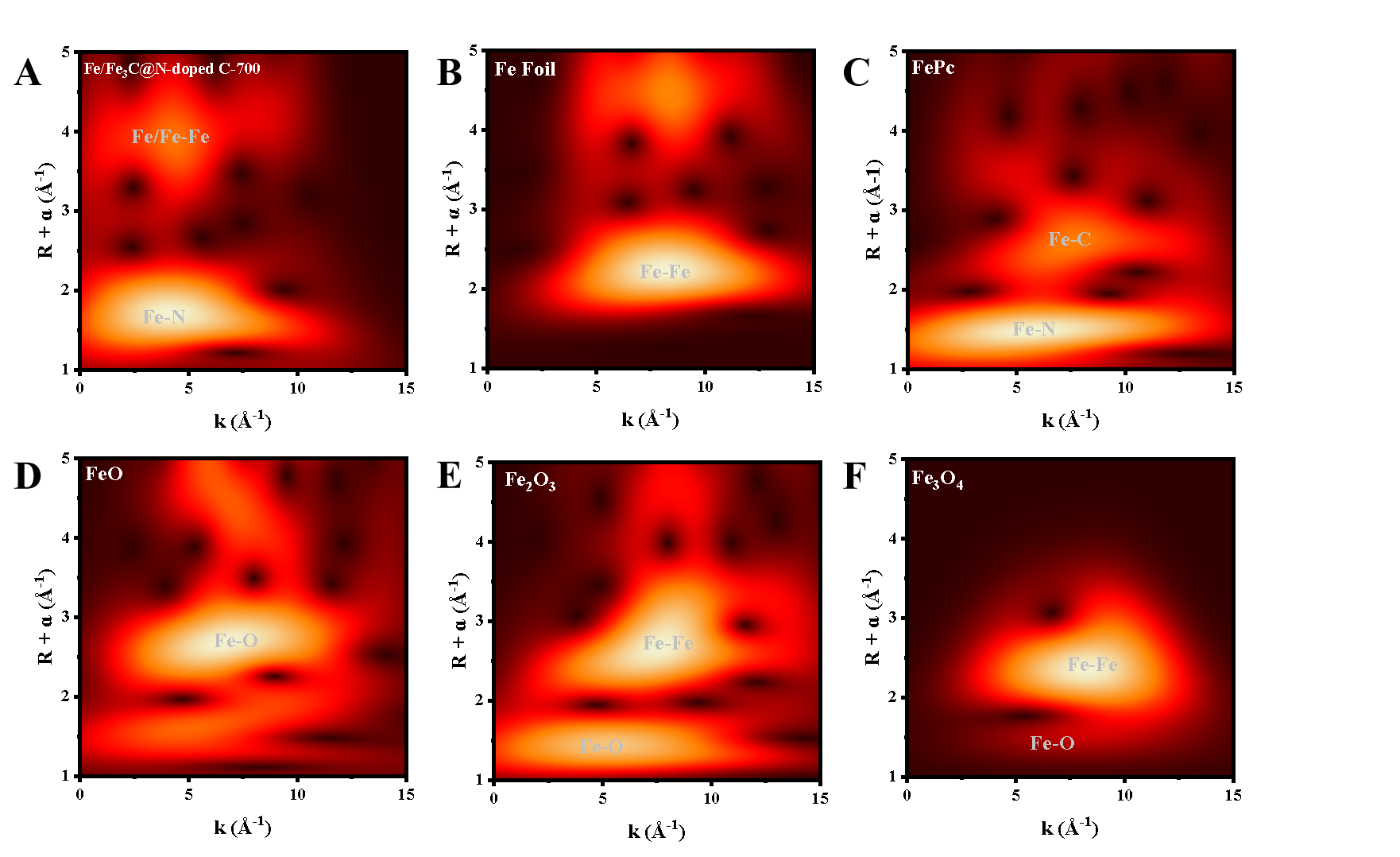


**Figure S10.** Wavelet transform of the k^3^-weighted EXAFS of Fe k-egde.


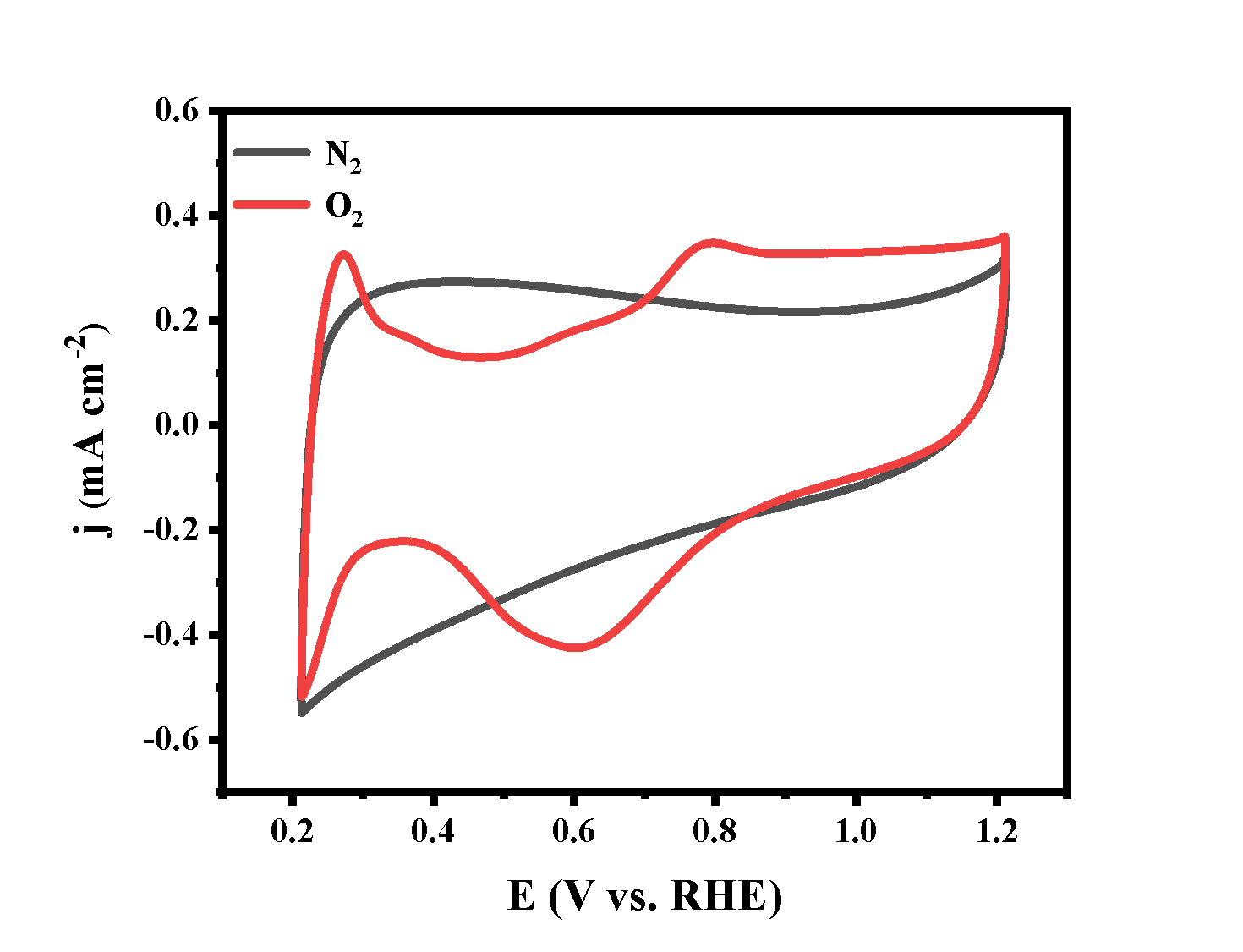


**Figure S11.** The cyclic voltammetry (CV) curves of Fe/Fe_3_C@N-doped C/CC-700 in the 0.1 M KOH electrolyte saturated with O_2_ and N_2_.


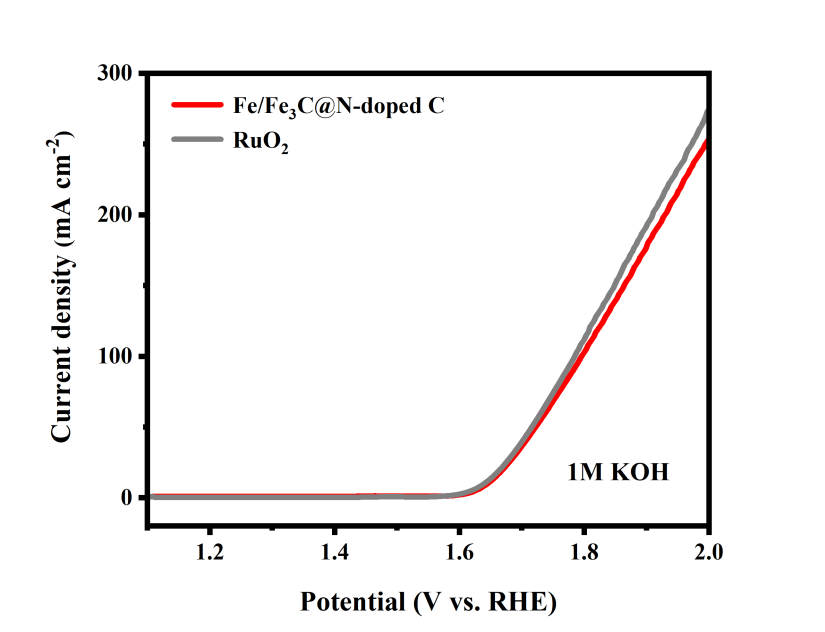


**Figure S12.** LSV curves in 1 M KOH at a scan rate of 10 mV s^-1^.


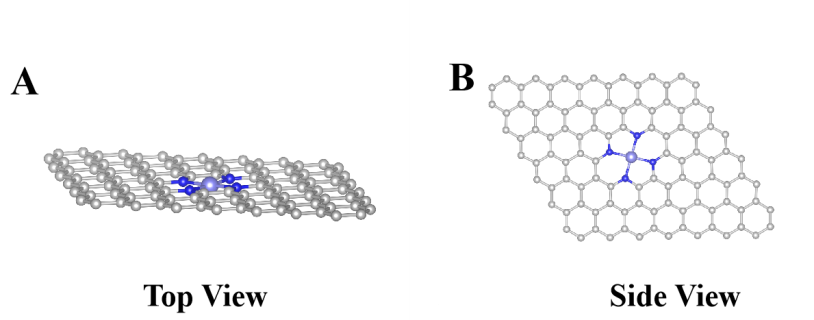


**Figure S13.** Computational Structure Model of Fe-N-C and corresponding A) top view and B) side view.


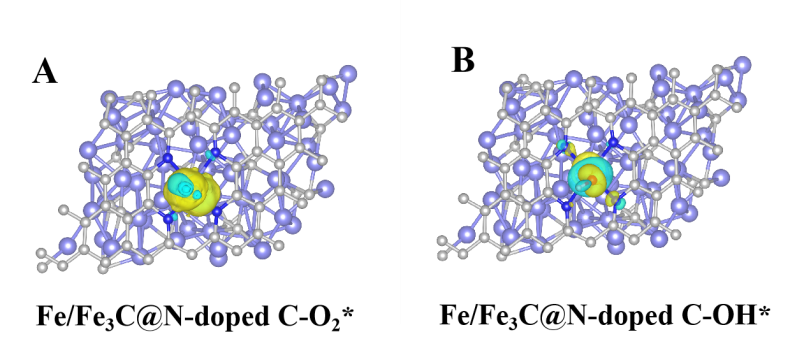


**Figure S14.** The top view of Fe/Fe_3_C@N-doped C electron difference density plots of A) O_2_* and B) OH* intermediate adsorption and desorption.


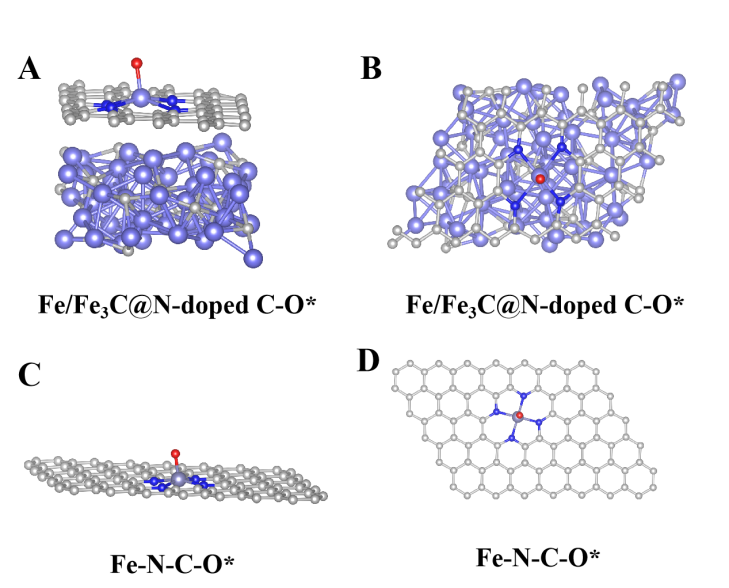


**Figure S15.** The top view and side view of O* adsorption of A-B) Fe/Fe_3_C@N-doped C and C-D) Fe-N-C.


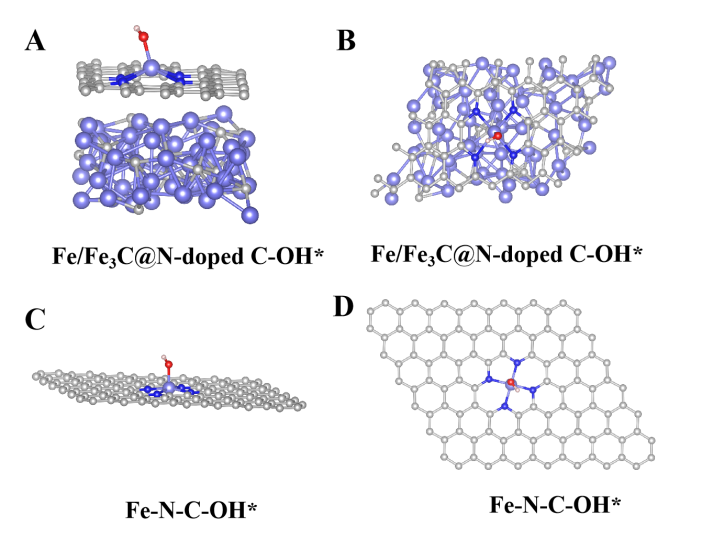


**Figure S16.** The top view and side view of O* adsorption of A-B) Fe/Fe_3_C@N-doped C and C-D) Fe-N-C.


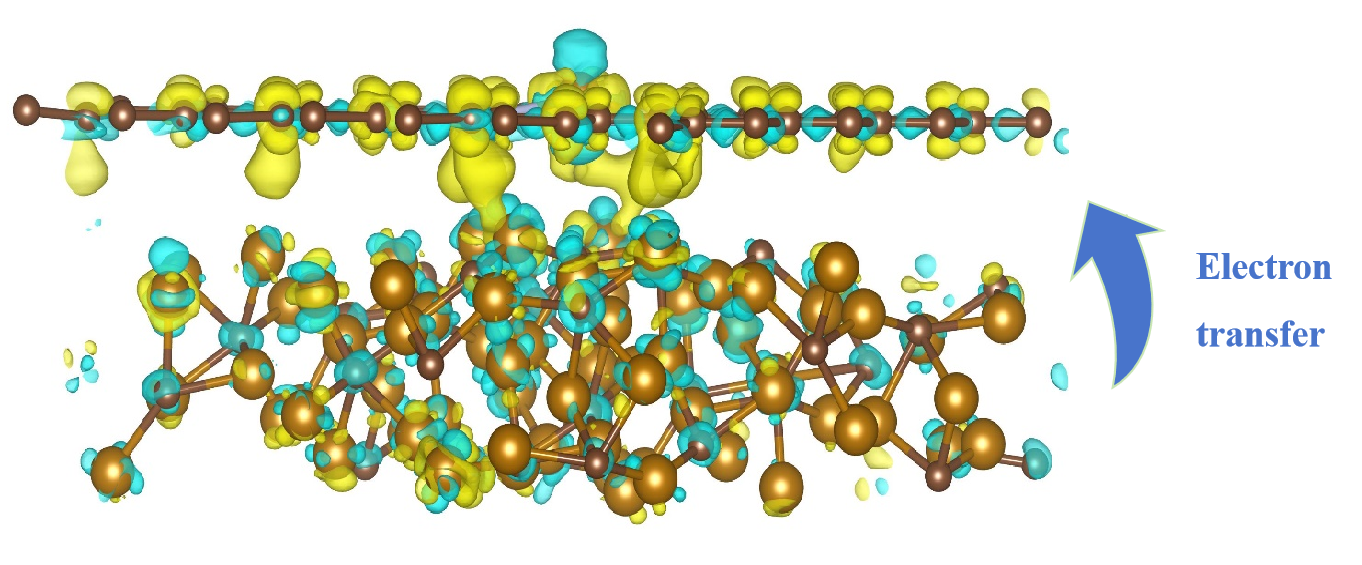


**Figure S17.** Charge density distributions for the Fe-N-C/Fe_3_C model.


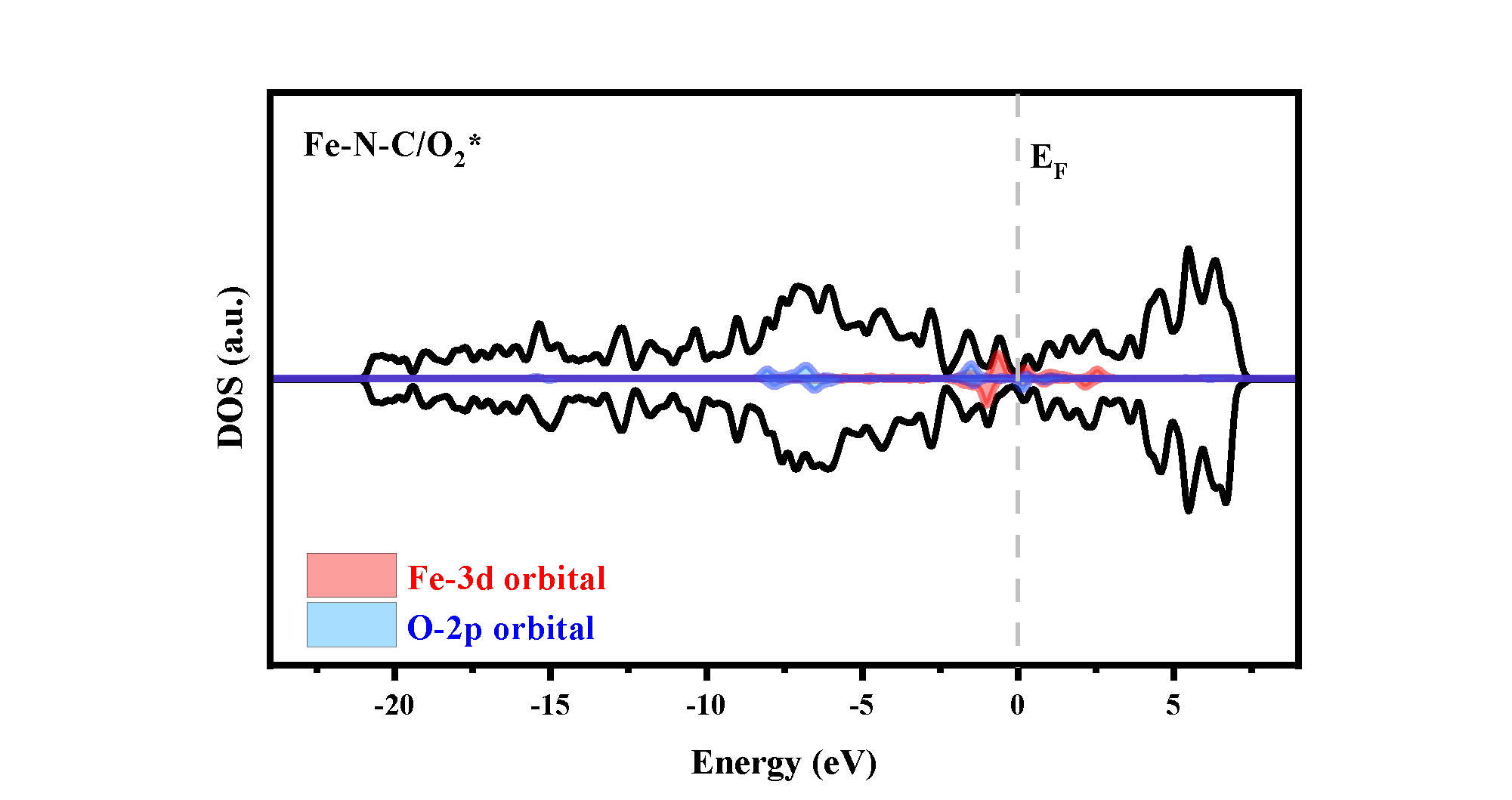


**Figure S18.** Fe-N-C for DOSs of O_2_* intermediates, where the Fermi level is represented by a gray dashed line.


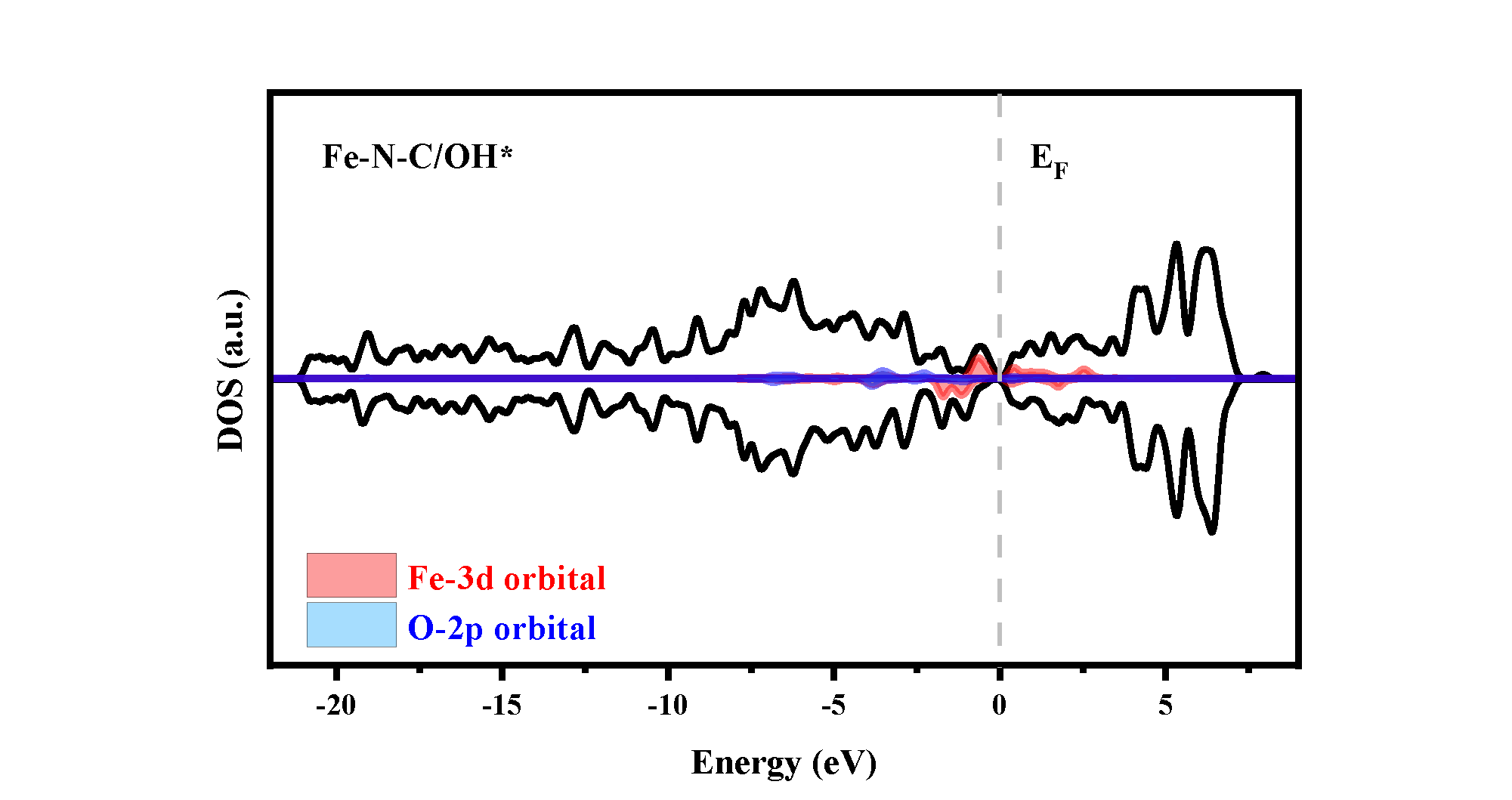


**Figure S19.** Fe-N-C for DOSs of OH* intermediates, where the Fermi level is represented by a gray dashed line.


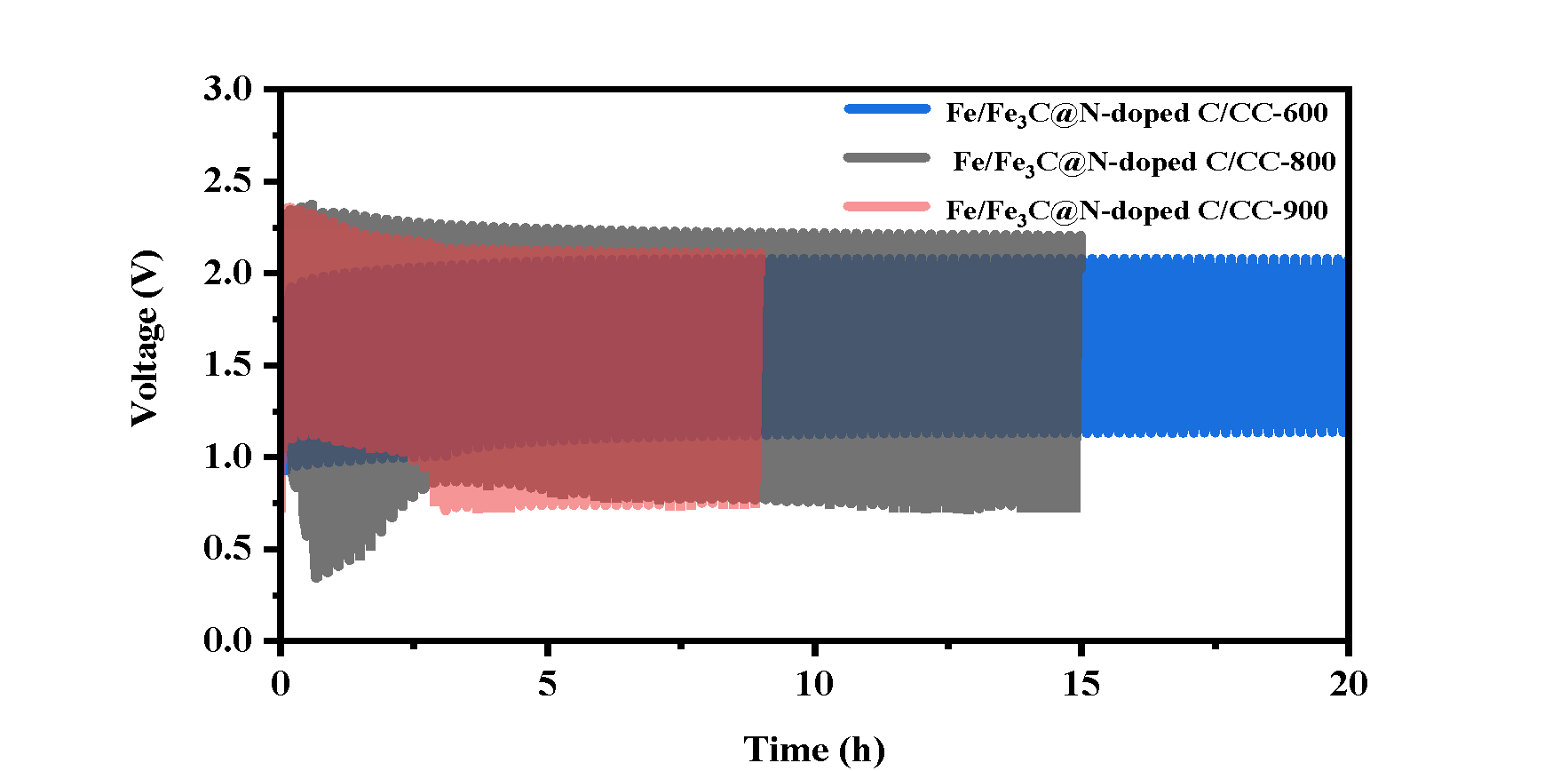


**Figure S20.** The cyclic performance of ZABs (10 mins charging and 10 mins discharging) for Fe/Fe_3_C@N-doped C/CC-600, Fe/Fe_3_C@N-doped C/CC-800, and Fe/Fe_3_C@N-doped C/CC-900 at a current density of 10 mA cm^-2^ under 20h.


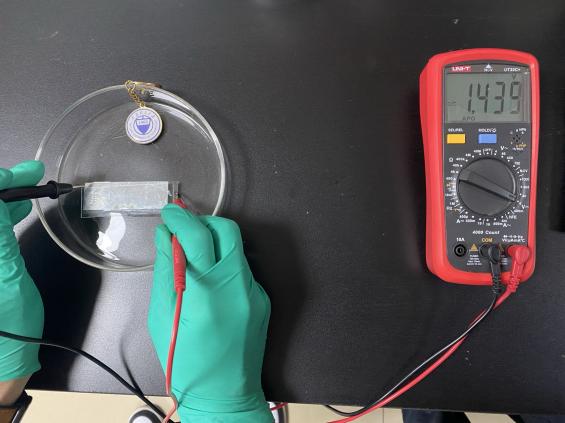


**Figure S21.** Fe/Fe_3_C@N-doped C/CC-700 test the open circuit voltage (OCV) directly as the FZABs cathode.


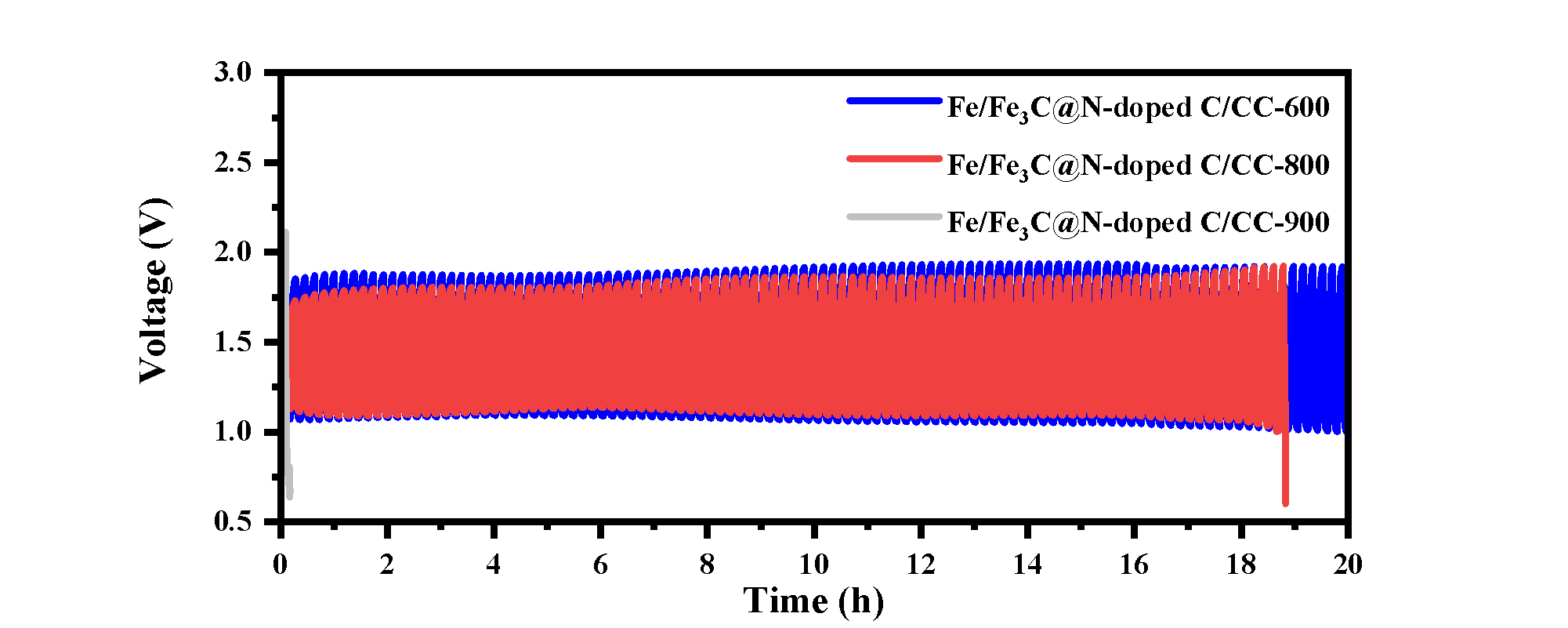


**Figure S22.** The cyclic performance of FZABs (10 mins charge and 10 mins discharge) at a current density of 5 mA cm^-2^.


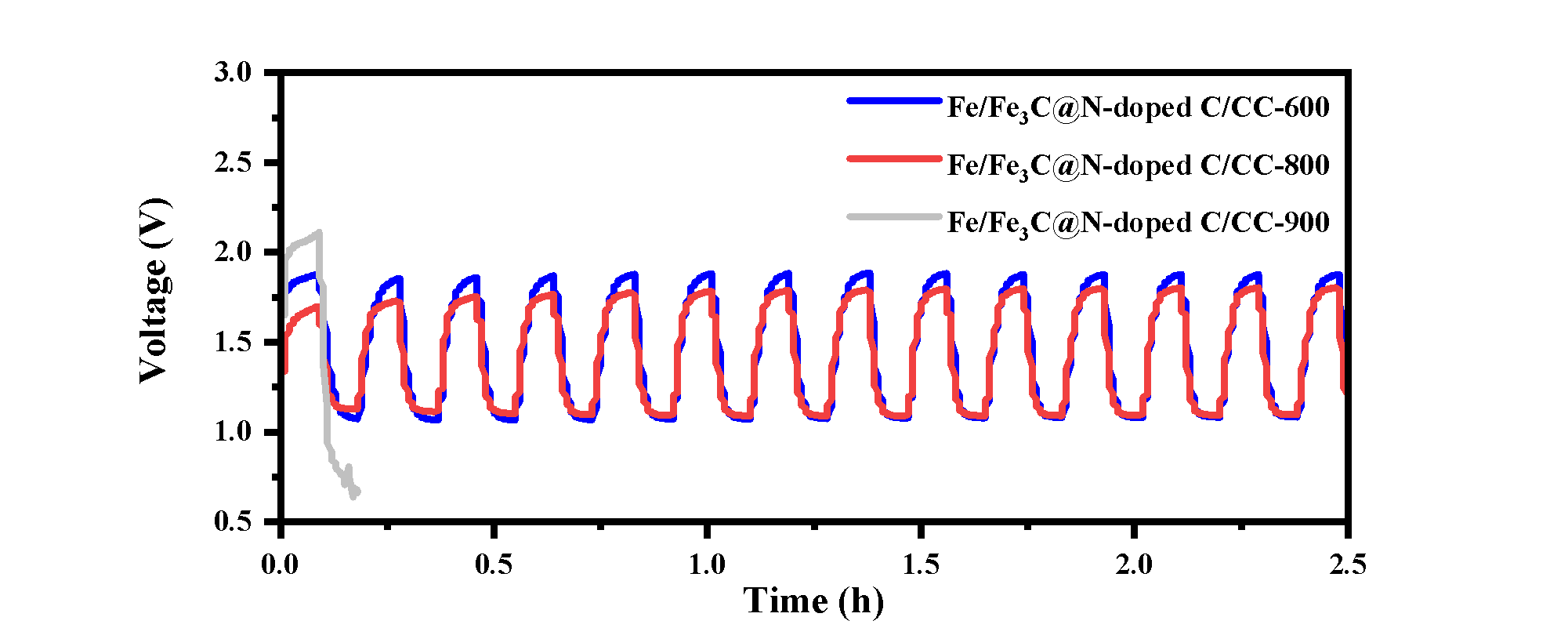


**Figure S23.** The cyclic performance of FZABs (10 mins charge and 10 mins discharge) at a current density of 5 mA cm^-2^ under 2.5h.


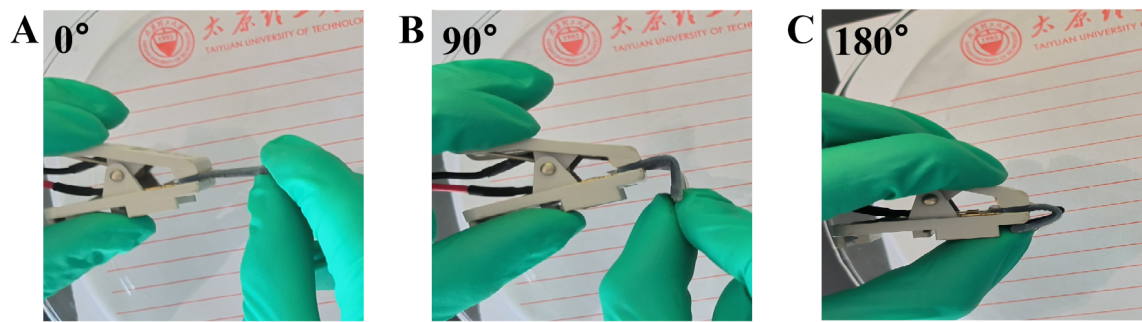


**Figure S24.** Different bending condition of FZABs under 0°, 90°, 180°.

**Table S1.** Element content in Fe/Fe_3_C@N-doped C/CC-700 and Fe/Fe_3_C@N-doped C/CC-Hydro structure measured by ICP.

| Catalyst materials | Fe/Fe_3_C@N-doped C/CC-700 | Fe/Fe_3_C@N-doped C/CC-Hydro |
| --- | --- | --- |
| Fe (wt%) | 3.14 | 0.53 |

**Table S2.** The calculated atomic ratio of different atoms in Fe/Fe_3_C@N-doped C/CC-700 based on XPS analysis.

| Catalyst materials | Fe (wt%) | N (wt%) | C (wt%) |
| --- | --- | --- | --- |
| Fe/Fe_3_C@N-doped C/CC-700 | 1.36 | 2.38 | 96.26 |

**Table S3.** Comparison of ORR performance of Fe/Fe_3_C@N-doped C/CC-700 and Fe/Fe_3_C@N-doped C/CC-600, Fe/Fe_3_C@N-doped C/CC-800 and Fe/Fe_3_C@N-doped C/CC-900.

| Catalysts  material | E (V) | | Tafel Slope (mV dec^-1^) |
| --- | --- | --- | --- |
|  | E_1/2_ | E_oneset_ | ORR |
| Fe/Fe_3_C@N-doped C/CC-700 | 0.903 | 1.04 | 75.4 |
| Fe/Fe_3_C@N-doped C/CC-600 | 0.809 | 1.07 | 101.7 |
| Fe/Fe_3_C@N-doped C/CC-800 | 0.791 | 1.10 | 136.9 |
| Fe/Fe_3_C@N-doped C/CC-900 | 0.729 | 0.91 | 124.5 |
| Pt/C | 0.874 | 1.01 | 89 |

**Table S4.** Summary of previously reported bifunctional catalysts in terms of their performance.

| Catalysts  material | E_1/2_  (V vs.RHE) in 0.1 M KOH | ORR  Tafel slope/mV dec^-1^ | Power density  (mW cm^-2^) | Ref. |
| --- | --- | --- | --- | --- |
| Fe/Fe_3_C@N-doped C/CC-700 | 0.903 | 75.4 | 160.5 | This work |
| Co-NC@LDH^[6]^ | 0.8 | 53.96 | 107.8 | *Chem. Eng. J.*  **2020**,  *399*,125718 |
| Ni,Fe-DSAs/NCs^[7]^ | 0.89 | 64 | 217.5 | *ACS Nano.*  **2023**,  *17*, 8622 |
| Fe_1_Co_1_-CNF^[8]^ | 0.87 | 88 | 201.7 | *Nano Energy.*  **2021**,  *87*, 106147 |
| H_2_PO_2_ - /FeNi-LDH-V_2_C^[9]^ | 0.8 | 52.8 | 137 | *Appl. Catal. B: Environ.*  **2021**,  *297*, 120474 |
| IrCo-N-C^[10]^ | 0.91 | 54 | NA | *ACS Catal*.  **2021**,  *11*, 8837 |
| Fe SAs HS^[11]^ | 0.86 | 90 | 170 | *Angew. Chem. Int. Ed.*  **2023**,  *62*, e202304229 |
| Ni-N_4_/GHSs/Fe-N_4_^[12]^ | 0.83 | 55 | NA | *Adv. Mater.*  **2020**,  *32*, 202003134 |
| Fe_1_Co_3_-NC-1100^[13]^ | 0.88 | 69.06 | 372 | *ACS Catal*.  **2022**,  *12*, 1216 |
| FeNiCo@NC-P^[14]^ | 0.84 | 53 | 112 | *Adv. Funct. Mater.* **2019**,  1908167 |
| Co-Nx-YSC-600/CC^[15]^ | 0.8 | 85.3 | NA | *Nano Energy*  **2021**,  *89*,106314 |
| Co_1_-PNC/Ni_1_-PNC^[16]^ | 0.88 | 63 | 252 | *Nano Res.*  **2021**,  *14(10)*: 3482 |
| CoNC SAC^[17]^ | 0.86 | 47.9 | 161.8 | *Sci. Adv.*  **2022**,  *8*, eabn5091 |

**Table S5.** Structural parameters at the Fe K-edge extracted from EXAFS fittings.

| Samples | Path | CN | R(Å) | σ^2^ (10^-3^Å^2^) | ΔE_0_ (eV) | R factor |
| --- | --- | --- | --- | --- | --- | --- |
| Fe foil | Fe-Fe | 4^*^ | 3.1229 | 0.66 | -57.308 | 0.0016 |
| FeO | Fe-Fe | 4 | 3.3917 | 18.87 | 3.393 | 0.0177 |
|  | Fe-O | 1 | 2.1132 | 8.0 | 3.393 |  |
| Fe_2_O_3_ | Fe-Fe | 2 | 2.9771 | 13.28 | -1.128 | 0.0161 |
|  | Fe-O | 3 | 2.0011 | 9.68 | -1.128 |  |
| Fe_3_O_4_ | Fe-Fe | 6 | 3.05910 | 244.48 | 0.034 | 0.0146 |
|  | Fe-O | 2 | 2.00852 | 10.97 | -0.086 |  |
| FePc | Fe-C | 4 | 2.81898 | 10.55 | 5.178 | 0.0170 |
|  | Fe-N | 2 | 1.96164 | 7.06 | 5.178 |  |
| Fe/Fe_3_C@N-doped C/CC-700 | Fe-N | 4 | 2.01162 | -0.10 | 10.027 | 0.0204 |
|  | Fe-C | 3 | 3.77758 | -6.45 | 10.027 |  |
|  | Fe-Fe | 2 | 2.50242 | 0.72 | 10.027 |  |

CN and R are the coordination number and bond length of atoms, respectively. σ^2^ represents Debye-Waller factor (a measure of thermal and static disorder in absorber

scatterer distances). ΔE_0_ is edge-energy shift (the difference between the zero kinetic energy value of the sample and that of the theoretical model). R factor displays the goodness of the fitting. * This value was fixed during EXAFS fitting. Error bounds of the structural parameters obtained from EXAFS fitting were about CN ± 20%, R ± 1%, σ^2^ ± 20% and ΔE_0_ ± 20%, respectively^[18]^.

**Reference**

1. G. Kresse, J. Hafner, *Phys. Rev. B*. **1993**, *47*, 558.
2. G. Kresse, J. Hafner, *Phys. Rev. B*. **1994**, *49*, 14251.
3. J. P. Perdew, K. Burke, M. Ernzerhof, *Phys. Rev. Lett*. **1996**, *77*, 3865.
4. G. Kresse, D. Joubert, *Phys. Rev. B*. **1999**, *59*, 1758.
5. P. E. Blöchl, *Phys. Rev. B*. **1994**, *50*, 17953.
6. D. Chen, X. Chen, Z. Cui, G. Li, B. Han, Q. Zhang, J. Sui, H. Dong, J. Yu, L. Yu, L. Dong, *Chem. Eng. J*. **2020**, *399*, 125718.
7. Z. Wang, X. Jin, R. Xu, Z. Yang, S. Ma, T. Yan, C. Zhu, J. Fang, Y. Liu, S. Hwang, Z. Pan, H. Fan, *ACS Nano*. **2023**, *17*, 8622.
8. Y. Wang, Z. Li, P. Zhang, Y. Pan, Y. Zhang, Q. Cai, S. Silva, J. Liu, G. Zhang, X. Sun, Z. Yan, *Nano Energy*. **2021**, *87*, 106147.
9. Y. Chen, H. Yao, F. Kong, H. Tian, G. Meng, S. Wang, X. Mao, X. Cui, X. Hou, J. Shi, *Appl. Catal. B: Environ*. **2021**, *297*, 120474.
10. M. Xiao, J. Zhu, S. Li, G. Li, W. Liu, Y. Deng, Z. Bai, L. Ma, M. Feng, T. Wu, D. Su, J. Lu, A. Yu, Z. Chen, *ACS Catal*. **2021**, *11*, 8837.
11. Y. Wang, P. Meng, Z. Yang, M. Jiang, H. Li, J. Zhang, B. Sun, C. Fu, *Angew. Chem. Int. Ed*. **2023**, *62*, e202304229.
12. J. Chen, H. Li, C. Fan, Q. Meng, Y. Tang, X. Qiu, G. Fu, T. Ma, *Adv. Mater*. **2020**, *32*, 202003134.
13. Y. He, X. Yang, Y. Li, L. Liu, S. Guo, C. Shu, F. Liu, Q. Tan, G. Wu, *ACS Catal*. **2022**, *12*, 1216.
14. D. Ren, J. Ying, M. Xiao, Y. Deng, J. Ou, J. Zhu, G. Liu, Y. Pei, S. Li, A. Jauhar, H. Jin, S. Wang, D. Su, A. Yu, Z. Chen, *Adv. Funct. Mater*. **2019**, *30*, 1908167.
15. Z. Li, J. Yang, X. Ge, Y. Deng, G. Jiang, H. Li, G. Sun, W. Liu, Y. Zheng, H. Dou, H. Jiao, J. Zhu, N. Li, Y. Hu, M. Feng, Z. Chen, *Nano Energy*. **2021**, *89*, 106314.
16. Y. Wang, X. Wan, J. Liu, W. Li, Y. Li, X. Guo, X. Liu, J. Shang, J. Shui, *Nano Res*. **2021**, *14*, 3482.
17. C. Zhao, J. Liu, J. Wang, C. Wang, X. Guo, X. Li, X. Chen, L. Song, B. Li, Q. Zhang, *Sci. Adv*, **2022**, *8*, eabn5091.
18. B. Ravel, M. Newville, *J. Synchrotron Radiat*. **2005**, *12*, 537.
